# Macrophage Response to Avirulent and Virulent *Mycobacterium tuberculosis* and Anti-TB Effects of Exosome Treatment

**DOI:** 10.1093/gpbjnl/qzaf065

**Published:** 2025-08-05

**Authors:** Li Yang, Lingna Lyu, Cuidan Li, Xiuli Zhang, Yingjiao Ju, Ju Zhang, Jie Liu, Liya Yue, Nan Ding, Xiangli Zhang, Dandan Lu, Tingting Yang, Peihan Wang, Jie Wang, Xiaotong Wang, Sihong Xu, Yongjie Sheng, Chunlai Jiang, Jing Wang, Xin Hu, Bahetibieke Tuohetaerbaike, Zongde Zhang, Fei Chen

**Affiliations:** China National Center for Bioinformation, Beijing 100101, China; Beijing Institute of Genomics, Chinese Academy of Sciences, Beijing 100101, China; University of Chinese Academy of Sciences, Beijing 101408, China; Department of Dermatology, Xiangya Hospital, Central South University, Changsha 410008, China; Department of Gastroenterology and Hepatology, Beijing Youan Hospital, Capital Medical University, Beijing 100069, China; Beijing Key Laboratory for Drug Resistant Tuberculosis Research, Beijing Chest Hospital, Capital Medical University, Beijing Tuberculosis and Thoracic Tumor Research Institute, Beijing 101149, China; China National Center for Bioinformation, Beijing 100101, China; Beijing Institute of Genomics, Chinese Academy of Sciences, Beijing 100101, China; China National Center for Bioinformation, Beijing 100101, China; Beijing Institute of Genomics, Chinese Academy of Sciences, Beijing 100101, China; University of Chinese Academy of Sciences, Beijing 101408, China; China National Center for Bioinformation, Beijing 100101, China; Beijing Institute of Genomics, Chinese Academy of Sciences, Beijing 100101, China; University of Chinese Academy of Sciences, Beijing 101408, China; China National Center for Bioinformation, Beijing 100101, China; Beijing Institute of Genomics, Chinese Academy of Sciences, Beijing 100101, China; Biomedical Innovation Center, Beijing Shijitan Hospital, Capital Medical University, Beijing 100038, China; China National Center for Bioinformation, Beijing 100101, China; Beijing Institute of Genomics, Chinese Academy of Sciences, Beijing 100101, China; University of Chinese Academy of Sciences, Beijing 101408, China; China National Center for Bioinformation, Beijing 100101, China; Beijing Institute of Genomics, Chinese Academy of Sciences, Beijing 100101, China; University of Chinese Academy of Sciences, Beijing 101408, China; China National Center for Bioinformation, Beijing 100101, China; Beijing Institute of Genomics, Chinese Academy of Sciences, Beijing 100101, China; Biomedical Innovation Center, Beijing Shijitan Hospital, Capital Medical University, Beijing 100038, China; China National Center for Bioinformation, Beijing 100101, China; Beijing Institute of Genomics, Chinese Academy of Sciences, Beijing 100101, China; University of Chinese Academy of Sciences, Beijing 101408, China; China National Center for Bioinformation, Beijing 100101, China; Beijing Institute of Genomics, Chinese Academy of Sciences, Beijing 100101, China; University of Chinese Academy of Sciences, Beijing 101408, China; China National Center for Bioinformation, Beijing 100101, China; Beijing Institute of Genomics, Chinese Academy of Sciences, Beijing 100101, China; University of Chinese Academy of Sciences, Beijing 101408, China; China National Center for Bioinformation, Beijing 100101, China; Beijing Institute of Genomics, Chinese Academy of Sciences, Beijing 100101, China; University of Chinese Academy of Sciences, Beijing 101408, China; China National Center for Bioinformation, Beijing 100101, China; Beijing Institute of Genomics, Chinese Academy of Sciences, Beijing 100101, China; University of Chinese Academy of Sciences, Beijing 101408, China; China National Center for Bioinformation, Beijing 100101, China; Beijing Institute of Genomics, Chinese Academy of Sciences, Beijing 100101, China; Division II of In Vitro Diagnostics for Infectious Diseases, Institute for In Vitro Diagnostics Control, National Institutes for Food and Drug Control, Beijing 102629, China; Key Laboratory for Molecular Enzymology and Engineering of Ministry of Education, School of Life Sciences, Jilin University, Changchun 130012, China; Key Laboratory for Molecular Enzymology and Engineering of Ministry of Education, School of Life Sciences, Jilin University, Changchun 130012, China; State Key Laboratory of Pathogenesis, Prevention and Treatment of High Incidence Diseases in Central Asia, Clinical Medicine Institute, The First Affiliated Hospital of Xinjiang Medical University, Urumqi 830013, China; State Key Laboratory of Pathogenesis, Prevention and Treatment of High Incidence Diseases in Central Asia, Clinical Medicine Institute, The First Affiliated Hospital of Xinjiang Medical University, Urumqi 830013, China; State Key Laboratory of Pathogenesis, Prevention and Treatment of High Incidence Diseases in Central Asia, Clinical Medicine Institute, The First Affiliated Hospital of Xinjiang Medical University, Urumqi 830013, China; Beijing Key Laboratory for Drug Resistant Tuberculosis Research, Beijing Chest Hospital, Capital Medical University, Beijing Tuberculosis and Thoracic Tumor Research Institute, Beijing 101149, China; China National Center for Bioinformation, Beijing 100101, China; Beijing Institute of Genomics, Chinese Academy of Sciences, Beijing 100101, China; University of Chinese Academy of Sciences, Beijing 101408, China; State Key Laboratory of Pathogenesis, Prevention and Treatment of High Incidence Diseases in Central Asia, Clinical Medicine Institute, The First Affiliated Hospital of Xinjiang Medical University, Urumqi 830013, China; Beijing Key Laboratory of Genome and Precision Medicine Technologies, Beijing 100101, China; Key Laboratory of Viral Pathogenesis & Infection Prevention and Control (Jinan University), Ministry of Education, Guangzhou 510632, China

**Keywords:** Tuberculosis, *Mycobacterium tuberculosis*, H37Rv/H37Ra, Virulence, Transcriptome

## Abstract

Tuberculosis (TB) has returned as the leading cause of death caused by a single infectious agent in 2023. Human macrophages and their secreted exosomes play important roles in combating invading *Mycobacterium tuberculosis* (*Mtb*). However, a comprehensive understanding of the mechanisms underlying immune regulation in *Mtb*-infected macrophages, as well as the packaging mechanisms and anti-TB effects of *Mtb*-treated exosomes, is still lacking. Here, we conducted comprehensive analyses of the macrophages infected with avirulent and virulent *Mtb* strains (H37Ra and H37Rv) and their exosomes through omics and phenotypic approaches. The results showed that H37Ra stimulated strong immune responses and apoptosis in macrophages to eliminate invading *Mtb,* while H37Rv induced severe necrosis and immune escape for survival. Interestingly, our results suggest that macrophages kill *Mtb* in an interferon-gamma (IFN-γ)-independent but compensatory way, highlighting the central role of IFN signaling pathway in anti-TB response. Moreover, we observed selective transport of host and *Mtb* RNAs from macrophages to exosomes. Notably, H37Ra-treated exosomes displayed a higher anti-TB effect than H37Rv-treated exosomes due to some enriched pro-inflammatory and immune escape-related *Mtb* proteins in these two exosomes, respectively. In conclusion, our findings shed new light on the immune mechanisms of macrophages in response to *Mtb* infection, offering a new TB treatment strategy and promising vaccine candidates.

## Graphical abstract

**Figure:**
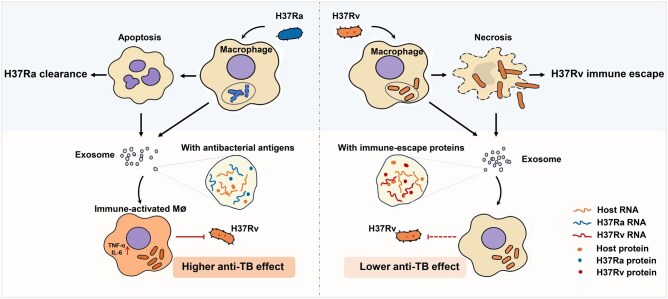

## Introduction

Tuberculosis (TB), caused by *Mycobacterium tuberculosis* (*Mtb*), regained its position as the world’s leading cause of death from a single infectious agent in 2023 [1–5]. According to the World Health Organization Global TB Report, 10.8 million people were diagnosed with TB in 2023, and 1.25 million died from the disease, reflecting a 4.5% increase from 2020 and reversing the previous two-decade trend of ∼ 2% annual declines [3].

The immune response to *Mtb* infection involves various immune cells [6–8]. Among them, macrophages are the most critical ones for protecting the host against *Mtb* through triggering multiple innate immune responses (including recognition, phagocytosis, and lysis of intracellular *Mtb*) [8]. Macrophages can also activate adaptive immunity to kill intracellular *Mtb* [including antigen presentation, CD4^+^ T cell activation, production of interferon-gamma (IFN-γ) and other important antibacterial cytokines, and further macrophage activation for phagocytosing/killing the invading *Mtb*] [9]. Several signaling pathways have been reported to play vital roles in regulating macrophage responses, such as Toll-like receptors, the autophagic signaling pathway, and the IFN signaling pathway [8].

Previous studies have explored the differential immune responses of macrophages infected with the virulent H37Rv and avirulent H37Ra strains of *Mtb*. Zhang et al. reported a faster growth rate of H37Rv than H37Ra in human monocyte-derived macrophages (hMDMs) [10]; Chen et al. found that H37Rv could cause necrosis by significant disruption of the mitochondrial inner membrane in hMDMs [11]; Freeman et al. demonstrated differential growth and cytokine/chemokine induction in H37Ra- and H37Rv-infected murine macrophages [12]. There are also several transcriptomic studies for the macrophages infected with H37Rv and H37Ra: Kalam et al. reported extensive remodeling of alternate splicing in the infected macrophage [13]; Lee et al. identified many differentially expressed genes (DEGs) related to innate immune responses in H37Ra- and H37Rv-infected hMDMs, and further confirmed the important role of solid carrier family 7 member 2 (*slc7a2*) in *Mtb* survival [14]; Pu et al. found that H37Rv infection could trigger off more severe inflammatory immune responses for facilitating tissue damage than H37Ra and *Bacillus calmette-guérin* (BCG) infections [15].

In recent years, exosomes have been reported to play important roles in various diseases, including TB [16]. Exosomes are extracellular, cup-shaped membrane vesicles (∼ 100 nm) that mediate signal transmission and substance transfer between cells by trafficking bioactive molecules (including DNA, RNA, proteins, and lipids) [17]. Secreted exosomes from infected cells are closely related to innate and acquired immune responses of the body through packing *Mtb* and human contents (including proteins and RNAs) [18,19]. Giri et al. identified 41 highly antigenic *Mtb* proteins in exosomes from H37Rv-infected J774 cells [20], and Obregón-Henao et al. showed that *Mtb* RNA in exosomes can induce apoptosis in human monocytes [21]. Our previous studies have also demonstrated the presence of various *Mtb* and host RNAs in serum exosomes from latent and active TB, many of which are related to immune responses [22,23].

Overall, human immune responses in various immune cells determine the outcome of *Mtb* infection, among which macrophages are the first defensive line against invading *Mtb*. However, a comprehensive analysis of the immune mechanisms in infected macrophages is still lacking. The second concern is to explore the differential immune responses of human macrophages in response to virulent H37Rv and avirulent H37Ra strains, and to investigate why H37Ra is cleared by macrophages while H37Rv survives. The third concern is to examine the mechanism by which host and *Mtb* RNAs are packaged into exosomes and to explore whether *Mtb*-treated exosomes can enhance macrophage resistance to infection and identify the genes responsible for this antibacterial effect.

## Results

### Differential survival outcomes of macrophages and invading *Mtb* between avirulent and virulent *Mtb* infections

**Figure 1:**
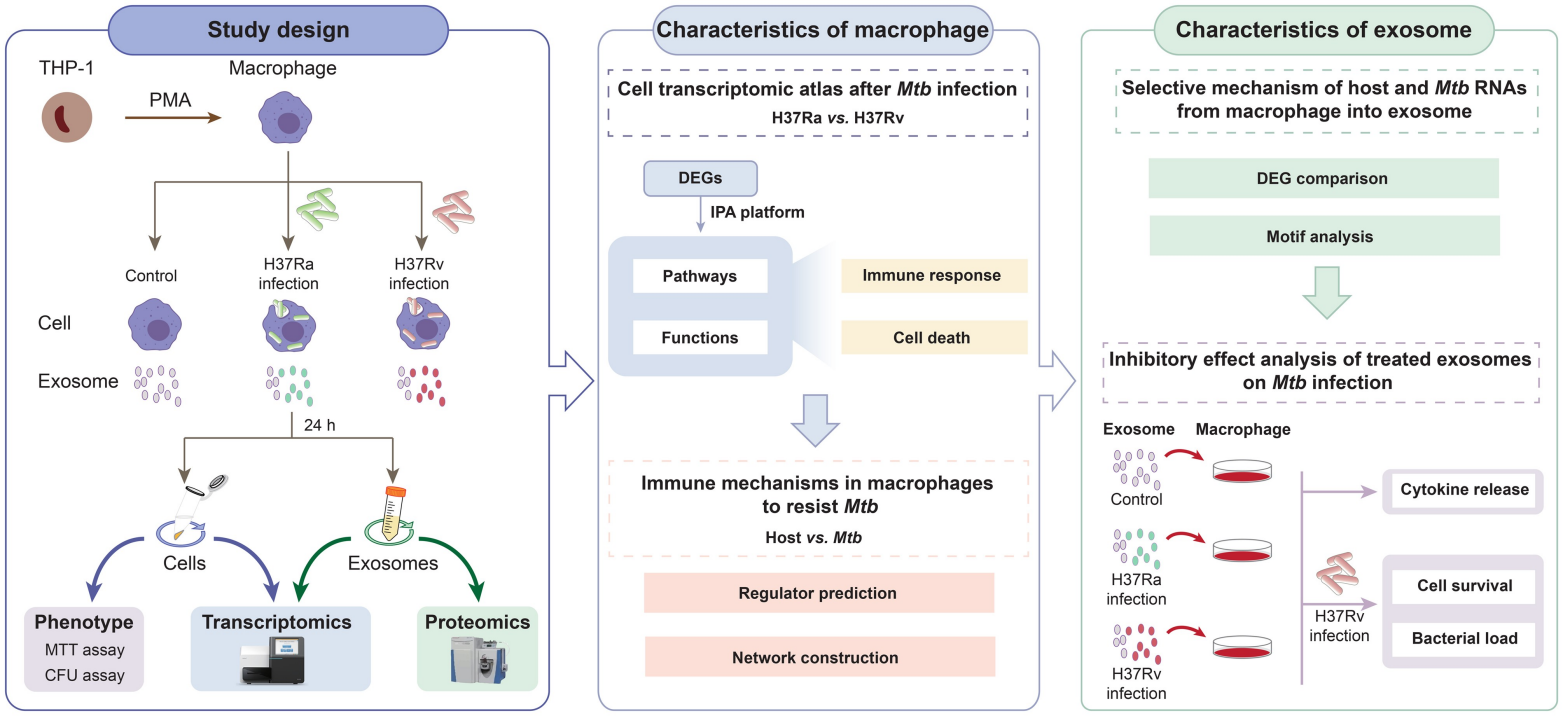
Diagram showing the schematic workflow including bioinformatic data analysis and experiments of this study Virulent H37Rv and avirulent H37Ra reference strains were used to infect PMA-differentiated THP-1 macrophages. Cells and corresponding exosomes were collected for phenotypic, transcriptomic, and proteomic studies (left panel). Characteristics of infected macrophages were further explored, including the transcriptomic atlas after *Mtb* infection and the immune mechanisms in macrophages to resist *Mtb* (middle panel). Characteristics of secreted exosomes were then analyzed, including the selective mechanisms and the anti-TB effect of the treated exosomes (right panel). PMA, phorbol 12-myristate 13-acetate; MTT, methyl thiazolyl tetrazolium; CFU, colony-forming unit; DEG, differentially expressed gene; IPA, Ingenuity Pathway Analysis; *Mtb*, *Mycobacterium tuberculosis*.

**Figure 2:**
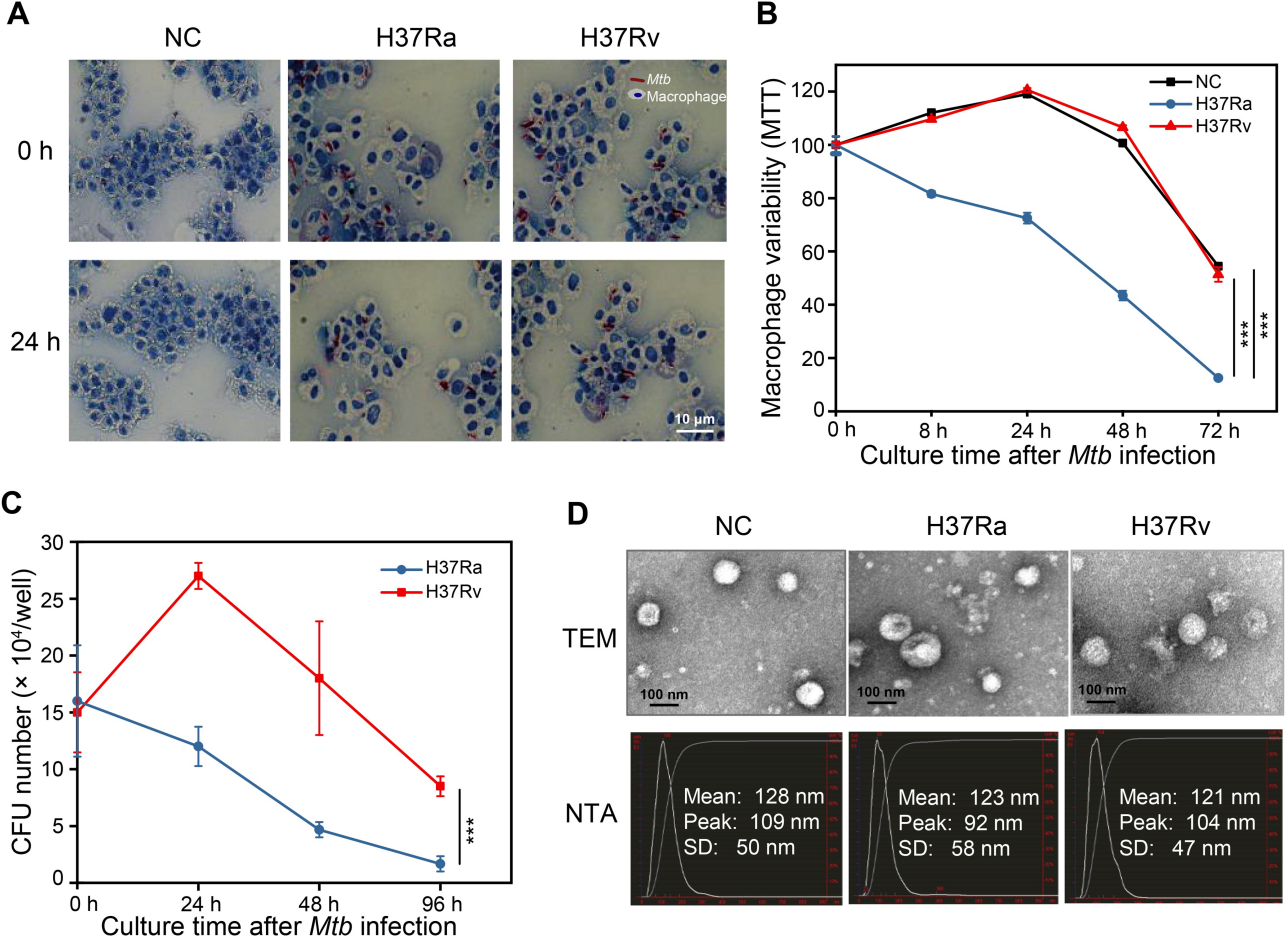
Differentially survival status of macrophages and invading *Mtb* between H37Ra and H37Rv infections **A**. Ziehl-Neelsen staining illustrating efficient *Mtb* infections in macrophages (MOI = 10) after 24 h. *Mtb* and cells are stained red and blue, respectively. **B**. MTT assay showing differential viability of macrophages infected with H37Ra and H37Rv (MOI = 10). The absorbance values are normalized to the uninfected control cells. **C**. CFU assay showing the viability of the invading *Mtb* in macrophages infected with H37Ra and H37Rv (MOI = 10). Data in (B) and (C) are from three independent experiments with biological replicates in each (mean ± SEM of *n* = 3 replicates). Statistical significance was determined by two-way ANOVA with Sidak’s multiple comparisons test. ***, *P* < 0.001. **D**. Exosome morphology and size distribution characterizations by TEM and NTA. The X-axis represents the size (diameter in nm), and the Y-axis represents the concentration (number of particles per ml). The red color highlights the scales of the X and Y axes, which we have added to the figure. Additionally, this figure shows three main values for the diameter of exosomes: the mean, peak, and SD (peak: ∼ 100 nm). MOI, multiplicity of infection; SEM, standard error of mean; SD, standard deviation; NTA, nanoparticle tracking analysis; TEM, transmission electron microscopy; NC, negative control.

To explore the infection mechanism of *Mtb* on human host cells, we infected phorbol 12-myristate 13-acetate (PMA)-differentiated THP-1 macrophages with the virulent H37Rv and avirulent H37Ra reference strains (**Figure 1**). Infection was confirmed for both strains through Ziehl-Neelsen (ZN) staining (**Figure 2**A). Methyl thiazolyl tetrazolium (MTT) and colony-forming unit (CFU) assays were conducted to measure macrophage viability and bacterial invasion, respectively (Figure 2B and C). The results showed different cell viability between macrophages infected with H37Rv and H37Ra. The growth of H37Rv-infected macrophages initially increased during the first 24 h, followed by a significant decline, similar to control macrophages. In contrast, H37Ra-infected macrophages exhibited continuous growth decline from the onset of infection (Figure 2B). Correspondingly, CFU assays showed differential survival rates of H37Rv and H37Ra. The bacterial load of H37Rv increased during the first 24 h of infection and then gradually decreased, while the bacterial load of H37Ra continuously decreased from the start (Figure 2C). These growth and survival patterns align with previous studies [24,25].

### Significant transcriptomic changes in macrophages and released exosomes after avirulent and virulent *Mtb* infections

Following 24 h of H37Rv and H37Ra infection, both macrophages and their released exosomes were collected for transcriptomic analysis. The purity and integrity of the exosomes were confirmed using transmission electron microscopy (TEM) and nanoparticle tracking analysis (NTA) (Figure 2D). Total RNAs were then extracted from the macrophage cells and exosomes, displaying characteristic cell and exosomal RNA profiles (Figure S1A) as previously reported [26]: cellular RNAs exhibited typical eukaryotic 5S, 18S, and 28S ribosomal RNA patterns, while exosomal RNAs showed a diffuse distribution with a high abundance of short RNA fragments.

**Figure 3:**
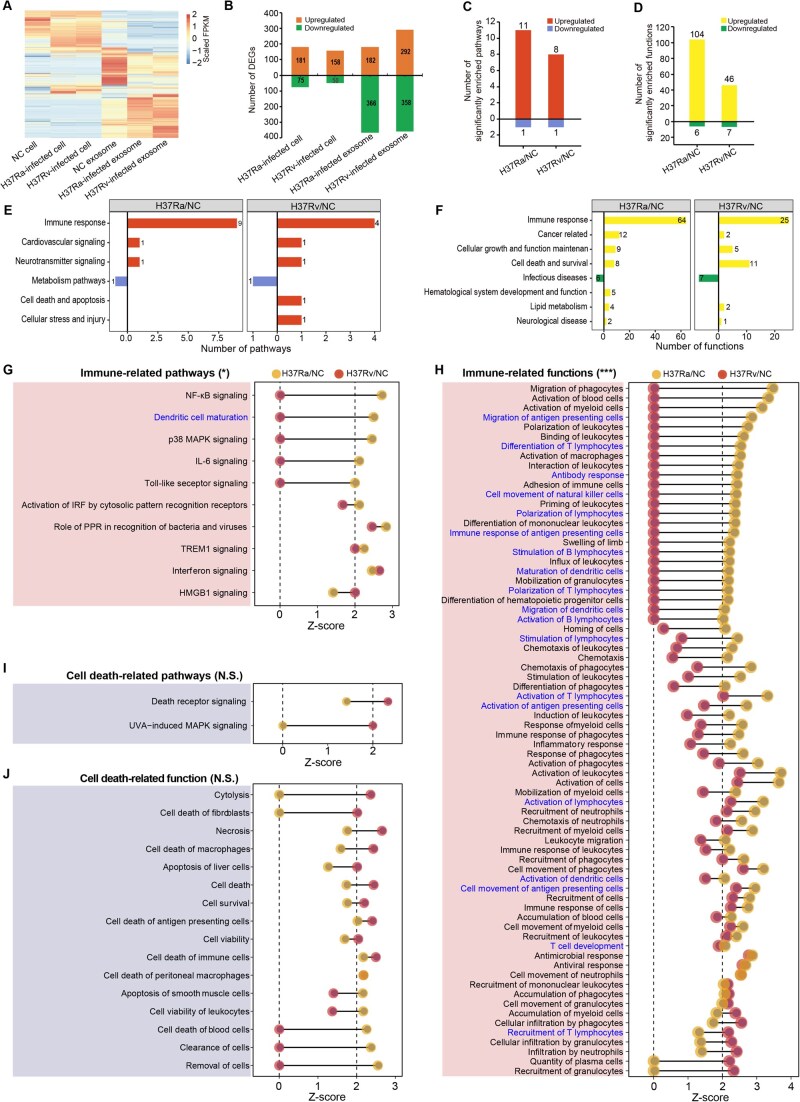
Expression profiles and significantly enriched pathways and biofunctions of the *Mtb*-infected macrophages and released exosomes **A**. Heatmap showing the DEGs among the six groups (|fold change| > 2, *P* < 0.05, *q* < 0.05). *P* values were calculated using a negative binomial model; *q* values were corrected by the Benjamini–Hochberg FDR procedure. **B**. Bar plot illustrating significantly upregulated/downregulated mRNAs in the *Mtb*-infected cells and released exosomes. Orange and green bars represent upregulated and downregulated mRNAs, respectively (|fold change| > 2, *P* < 0.05). *P* values were calculated using a negative binomial model. **C**. Bar plots demonstrating significantly enriched pathways in the H37Rv/NC and H37Ra/NC groups. **D**. Bar plots showing significantly enriched biofunctions in the H37Rv/NC and H37Ra/NC groups. **E**. Bar plots displaying the significantly enriched pathways in six aspects in the H37Rv/NC and H37Ra/NC groups. Red and blue bars indicate the significantly upregulated/downregulated pathways (Z-score > 2, *P* < 0.05), respectively. **F**. Bar plots illustrating the significantly enriched biofunctions in eight aspects in the H37Rv/NC and H37Ra/NC groups. Yellow and green bars represent the significantly upregulated/downregulated biofunctions (Z-score < −2, *P* < 0.05), respectively. Pathway and biofunction enrichment significance in (C)–(F) was determined by Fisher’s exact test. **G**. Dumbbell charts showing the innate and adaptive immunity pathways between the two groups. Blue font represents adaptive immunity pathways. **H**. Dumbbell charts displaying the innate and adaptive immunity biofunctions between the two groups. **I**. Dumbbell charts presenting the cell apoptosis/death related pathways between the two groups. **J**. Dumbbell charts showing the cell apoptosis/death related functions between the two groups. Statistical comparisons between the two groups were performed using the two-tailed paired Student’s *t*-test. N.S., not significant; *, *P* < 0.05; **, *P* < 0.01; ***, *P* < 0.001. mRNA, messenger RNA; FPKM, fragments per kilobase of transcript per million mapped reads.

Transcriptomic sequencing was then conducted on both infected macrophage cells and their secreted exosomes (Figure S1B and C). Compared to controls, both avirulent and virulent *Mtb* induced significant transcriptomic changes in the macrophages and exosomes (**Figure 3**A and B): 208 and 650 DEGs were identified in H37Rv-infected cells and their released exosomes, respectively; 256 and 548 DEGs were identified in the H37Ra-infected cells and their released exosomes, respectively (fold change > 2, *P* < 0.05).

### Immune response and cell death induced by avirulent and virulent *Mtb* in macrophages

To explore the immune mechanisms of human macrophages in response to *Mtb* infection, we analyzed significantly enriched pathways and biofunctions using Ingenuity Pathway Analysis (IPA). Compared to controls, most enriched pathways and biofunctions were significantly upregulated (*P* < 0.05, Z-score > 2): 11 out of 12 (91.67%) and 8 out of 9 (88.89%) pathways were significantly upregulated in the H37Ra/NC and H37Rv/NC groups, respectively, and only one pathway (PPAR signaling) was significantly downregulated in both groups (Figure 3C). Similarly, 104 of 110 (94.55%) and 46 of 53 (86.79%) biofunctions were significantly upregulated, with only six and seven downregulated biofunctions in the H37Ra and H37Rv groups, respectively (Figure 3D). Furthermore, these pathways and biofunctions were categorized into six and eight aspects, respectively (Figure 3E and F), with immune response-related pathways ranking highest in both groups. This suggests that macrophages activate multiple immune pathways to resist *Mtb* infection.

Notably, more immune response-related pathways and functions with Z-scores greater than 2, or those with higher Z-scores when activated in both groups, were detected in H37Ra-infected macrophages compared to those infected with H37Rv. This suggests a stronger immune response to avirulent *Mtb* and an immune escape strategy employed by virulent *Mtb* (Figure 3E–H), consistent with the phenotypic differences observed (Figure 2B and C). Among the 10 most significantly activated immune-related pathways (Figure 3G), six were specifically upregulated in the H37Ra group, including four innate immune pathways (*e.g.*, toll-like receptor signaling and NF-κB signaling) and two acquired immune response pathways (*e.g.*, dendritic cell maturation and IL-6 signaling). Shared pathways, such as “role of pattern recognition receptors in recognition of bacteria and viruses” and “TREM1 signaling”, were more activated in the H37Ra group than in the H37Rv group (Z-score: 2.828 *vs*. 2.449 and 2.236 *vs*. 2.000, respectively). Interestingly, the “IFN signaling” pathway was similarly activated in both groups (Z-score: 2.449 *vs*. 2.646). Among the 10 pathways, “HMGB1 signaling” was uniquely activated in H37Rv-infected macrophages.

Among the 72 enriched immune-related biofunctions (Figure 3H), 65.3% (47/72) were specifically activated in the H37Ra group. These included 32 innate immune functions (*e.g.*, phagocytosis, migration, and chemotaxis) and 15 acquired immune functions (*e.g.*, T/B lymphocyte activation). Shared biofunctions were also more strongly activated in the H37Ra group, with 82.35% (14/17) showing greater activation, including 11 innate and three acquired immune-related functions.

In contrast, non-apoptotic programmed cell death-related pathways and biofunctions were more activated in H37Rv-infected macrophages (Figure 3I and J). Enriched pathways related to cell deterioration and necrosis were specifically upregulated in the H37Rv group (Figure 3I). Among the 16 cell death-related biofunctions, 8 were uniquely activated in the H37Rv group, including “cytolysis”, “necrosis”, and “cell death” (Figure 3J). Two shared functions, “cell death of antigen-presenting cells” and “cell death of immune cells”, were more activated in the H37Rv group. These results suggest that H37Rv induces more severe non-apoptotic programmed cell death in macrophages. Incidentally, one apoptotic cell death-related function was specifically activated in H37Ra-infected macrophages (apoptosis of smooth muscle cells).

We also identified the top 10 upregulated DEGs in H37Ra- and H37Rv-infected samples (**Table 1**). Eight genes were common to both groups. Among these, five were related to immune response, including three cytokines (*CXCL8*, *CCL3*, and *CCL3L3*) and two innate immune genes (*IFI6* and *BST2*). Four immune-related DEGs (including the three cytokines and *IFI6*) showed higher expression in H37Ra-infected macrophages. Additionally, two DEGs (*FTL* and *FTH1*) specifically upregulated in H37Ra-infected macrophages have been linked to the production of reactive oxygen species (ROS) for bacterial clearance [27], while one DEG (*TDO2*) upregulated in H37Rv-infected macrophages is associated with immune evasion [28]. Comparing apoptosis-related and immune escape-related DEGs revealed that H37Ra upregulated more apoptosis-related genes (16/9) and fewer immune escape-related genes (4/9) (Table S1).

**Table 1:** The top 10 DEGs in the H37Ra- and H37Rv-infected macrophages

Gene name	FPKM (H37Ra/NC)	Fold change (H37Ra/NC)	Top 10 (H37Ra/NC)	FPKM (H37Rv/NC)	Fold change (H37Rv/NC)	Top 10 (H37Rv/NC)	Function
*FTL* ^#^	15,757.200	2.094	√	–	–	–	Production of reactive oxygen species
*CXCL8*	5729.650	4.453	√	5193.770	4.037	√	Production of reactive oxygen species, cell-to-cell signaling and interaction, cell death, and survival
*FTH1* ^#^	4884.620	2.036	√	–	–	–	Production of reactive oxygen species, cell death, and survival
*CCL3*	3088.310	4.919	√	1633.120	2.601	√	Cell death and survival, cell-to-cell signaling, and interaction
*CCL3L3*	2493.870	6.186	√	1379.520	3.423	√	Cell-to-cell signaling and interaction
*IFI6*	2197.590	6.519	√	2143.590	6.359	√	Innate immune response, cell death, and survival
*ISG15*	940.247	22.608	√	1275.980	30.681	√	Cell death and survival
*MFSD2A*	729.472	3.689	√	541.615	2.739	√	Cell-to-cell signaling and interaction
*RGS1*	631.227	2.647	√	716.261	3.004	√	Cell-to-cell signaling and interaction
*BST2*	630.502	2.871	√	718.411	3.272	√	Innate immune response
*TDO2* ^*^	–	–	–	591.311	2.681	√	Cell death and survival
*LY6E* ^*^	–	–	–	403.049	6.829	√	Cell-to-cell signaling and interaction

*Note*: ^#^, * represent the specifically enriched DEGs in the H37Ra- and H37Rv-infected macrophages, respectively. DEG, differentially expressed gene; FPKM, fragments per kilobase of transcript per million mapped reads; NC, negative control.

These findings demonstrate the distinct immune mechanisms activated in macrophages in response to H37Ra and H37Rv infections. H37Ra triggers a robust immune response and apoptotic cell death to eliminate *Mtb*, while H37Rv promotes immune escape and non-apoptotic cell death to facilitate its survival within macrophages.

### Compensatory activation of IFN signaling pathways in macrophages against *Mtb* infection through activation of downstream genes of IFNs

To further investigate the immune mechanisms in macrophages, we used IPA to predict upstream regulators of the DEGs. Interestingly, among the top 25 predicted upstream regulators (Tables S2 and S3), 3 were IFNs: IFN-γ (type II), IFN-α, and IFN-β1 (type I). Notably, IFN-γ was ranked as the top upstream regulator in both H37Ra- and H37Rv-infected macrophages (Z-score = 6.194 for H37Ra; Z-score = 5.698 for H37Rv). However, IFN-γ is typically produced by activated CD4^+^ T cells and natural killer (NK) cells, not macrophages [29], which was also confirmed by our transcriptomic data showing minimal expression of IFNs in infected macrophages (Table S4).

**Figure 4:**
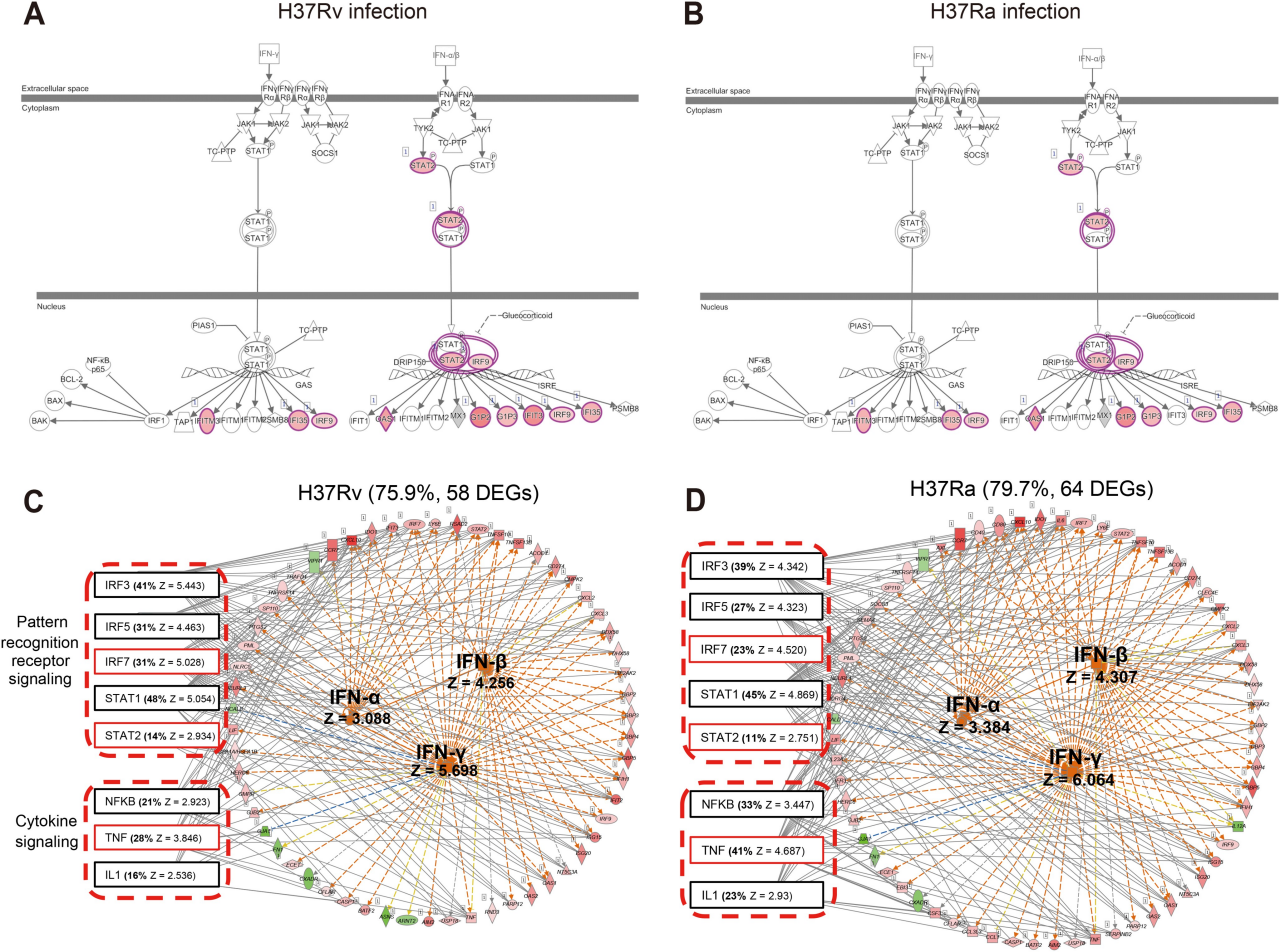
Compensatory activation of IFN signaling pathways in macrophages through activating the downstream genes of IFNs **A**. The activation of IFN signaling pathway after H37Rv infection. **B**. The activation of IFN signaling pathway after H37Ra infection. Pink circles represent molecules that match differential genes, and filled colors represent fold change. **C**. IFN pathway simulation network of H37Rv infection. **D**. IFN pathway simulation network of H37Ra infection. The IFNs in the circle are predicted upstream regulators, and the circular arrangement of molecules on the right represents the downstream DEGs that support IFN activation. The left eight predicted upstream regulators in dashed boxes represent the target molecules in simulation networks that have common downstream DEGs for IFN activation. IFN, interferon.

What caused the discrepancy between predicted upstream regulators and actual transcriptomic data regarding IFNs? We first observed significant activation of the “IFN signaling” pathways in both H37Ra- and H37Rv-infected macrophages (Figure 3G), likely due to the upregulation of downstream DEGs in these pathways, including seven DEGs in the Type I IFN signaling pathway (*STAT2*, *IRF9*, *OAS1*, *G1P2*, *G1P3*, *IFIT3*, and *IFI135*) and three DEGs (*IFITM3*, *IFI35*, and *IRF9*) in Type II IFN signaling pathway (**Figure 4**A and B). Thus, while the IFNs themselves were hardly expressed in our infection model, they were predicted as upstream regulators because of the activation of downstream genes in the IFN pathways.

Further analysis revealed that many activated downstream molecules were involved in all three IFN signaling pathways (IFN-α, IFN-β, and IFN-γ), including 64 and 58 DEGs in H37Ra- and H37Rv-infected macrophages, respectively (Figure 4C and D). However, since the three IFNs were hardly expressed in our *Mtb* infection model, we then searched for possible upstream regulatory genes to activate these downstream molecules. Here, the top eight predicted upstream regulatory factors were responsible for the activation of most of these downstream molecules, accounting for 79.7% (51/64) and 75.9% (44/58) of the H37Ra- and H37Rv-infected macrophages, respectively. Among these eight upstream factors, five were from the “pattern recognition receptor related signaling” pathway, and three were from the “cytokine signaling” pathway.

In summary, although the IFNs were hardly expressed in the transcriptomic data, the IFN signaling pathways in macrophages were activated in a compensatory manner in response to *Mtb* infection (Figures 3G, 4A, and 4B). This activation occurred through the downstream molecules being triggered by other upstream regulatory factors (Figure 4C and D). Our findings suggest that macrophages can combat *Mtb* via an IFN-γ-independent mechanism, but still rely on the activation of the IFN signaling pathway, underscoring its critical role in the anti-TB immune response.

### Selective secretion of characteristic host RNAs from *Mtb*-infected macrophages into exosomes

**Figure 5:**
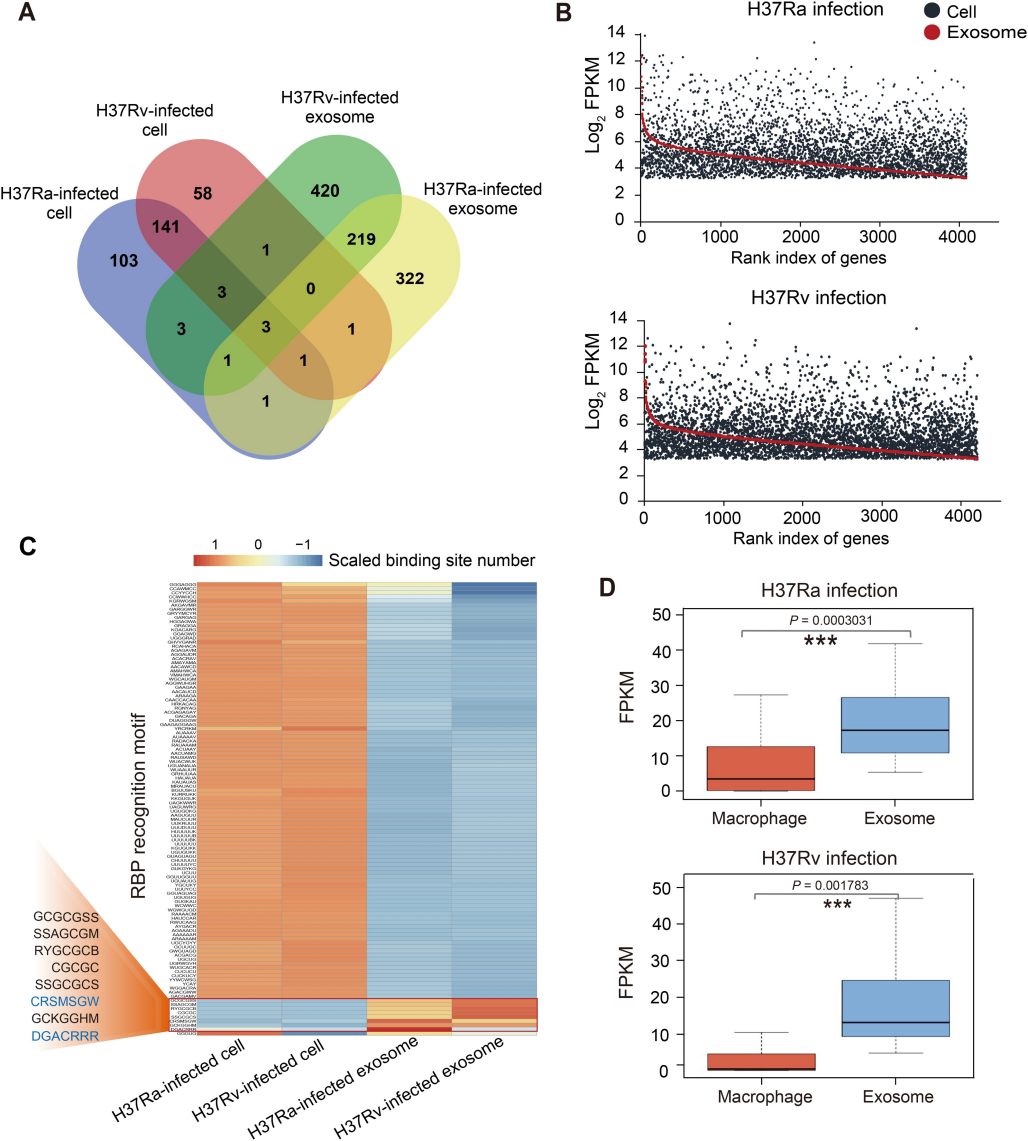
Selective secretion of characteristic host RNAs with RBP recognition motifs from *Mtb*-infected macrophages to exosomes **A**. Venn diagram showing the shared genes among the *Mtb*-infected cells and released exosomes (|fold change| > 2, *P* < 0.05, *q* < 0.05). *P* values were calculated using a negative binomial model; *q* values were corrected by the Benjamini–Hochberg FDR procedure. **B**. The abundance of all shared expressed genes (FPKM > 10: 4190 genes for Ra infection and 4194 genes for Rv infection) in the *Mtb*-infected cells and their secreted exosomes. **C**. RBP recognition motifs of the *Mtb*-infected cells and their released exosomes. Eight motifs are enriched in the exosomes (GCGCGSS, SSAGCGM, RYGCGCB, CGCGC, SSGCGCS, CRSMSGW, GCKGGHM, and DGACRRR). **D**. Boxplot showing the expressions of the genes with the above-mentioned eight motifs in the *Mtb*-infected macrophages and corresponding exosomes. Statistical comparisons were performed using the two-tailed paired Student’s *t*-test. ***, *P* < 0.001. RBP, RNA-binding protein.

To identify which host RNAs are selectively secreted into exosomes by *Mtb*-infected macrophages, we compared DEGs between infected macrophages and their exosomes (**Figure 5**A). The analysis revealed significant differences in RNA profiles between the two groups, indicating selective secretion of specific RNAs. Only seven and six DEGs were shared between H37Rv- or H37Ra-infected cells and their corresponding exosomes, representing approximately 1% of all exosomal DEGs (7/650 for H37Rv, 6/548 for H37Ra). Additionally, the expression levels of these shared genes in infected macrophages [4090/4194 genes in H37Ra and H37Rv, fragments per kilobase of transcript per million mapped reads (FPKM) > 10] were poorly correlated with their abundance in exosomes (Figure 5B).

To further investigate the RNA transport mechanism, we analyzed the RNA-binding protein (RBP) recognition motifs using the RBPmap database (Figure 5C) [30]. We found a differential distribution of RBP motifs between infected cells and their exosomes: most RBPs were enriched in RNAs from infected macrophages, while eight motifs associated with RNA binding, transport, and translocation were enriched in exosomal RNAs (Table S5) [27]. Genes containing these eight motifs showed higher abundance in exosomes than in the cells (Figure 5D), suggesting selective secretion via RBPs. Gene Ontology (GO) analysis revealed that these genes were involved in cell adhesion (Figure S2), consistent with their role in intercellular transport and signal transduction [18,19].

Further analysis showed differential distributions of the eight exosome-enriched motifs between virulent and avirulent *Mtb* infections (Figure 5C), indicating distinct RNA transport mechanisms. Two motifs (CRSMSGW and DGACRRR) were more abundant in exosomal RNA from H37Ra-infected macrophages, while five motifs (GCGCGSS, SSAGCGM, RYGCGCB, CGCGC, and SSGCGCS) were significantly enriched in exosomes from H37Rv-infected macrophages. The motif gckgghm showed comparable abundance under both conditions.

### Directed transport of *Mtb* RNAs to exosomes from macrophages

**Figure 6:**
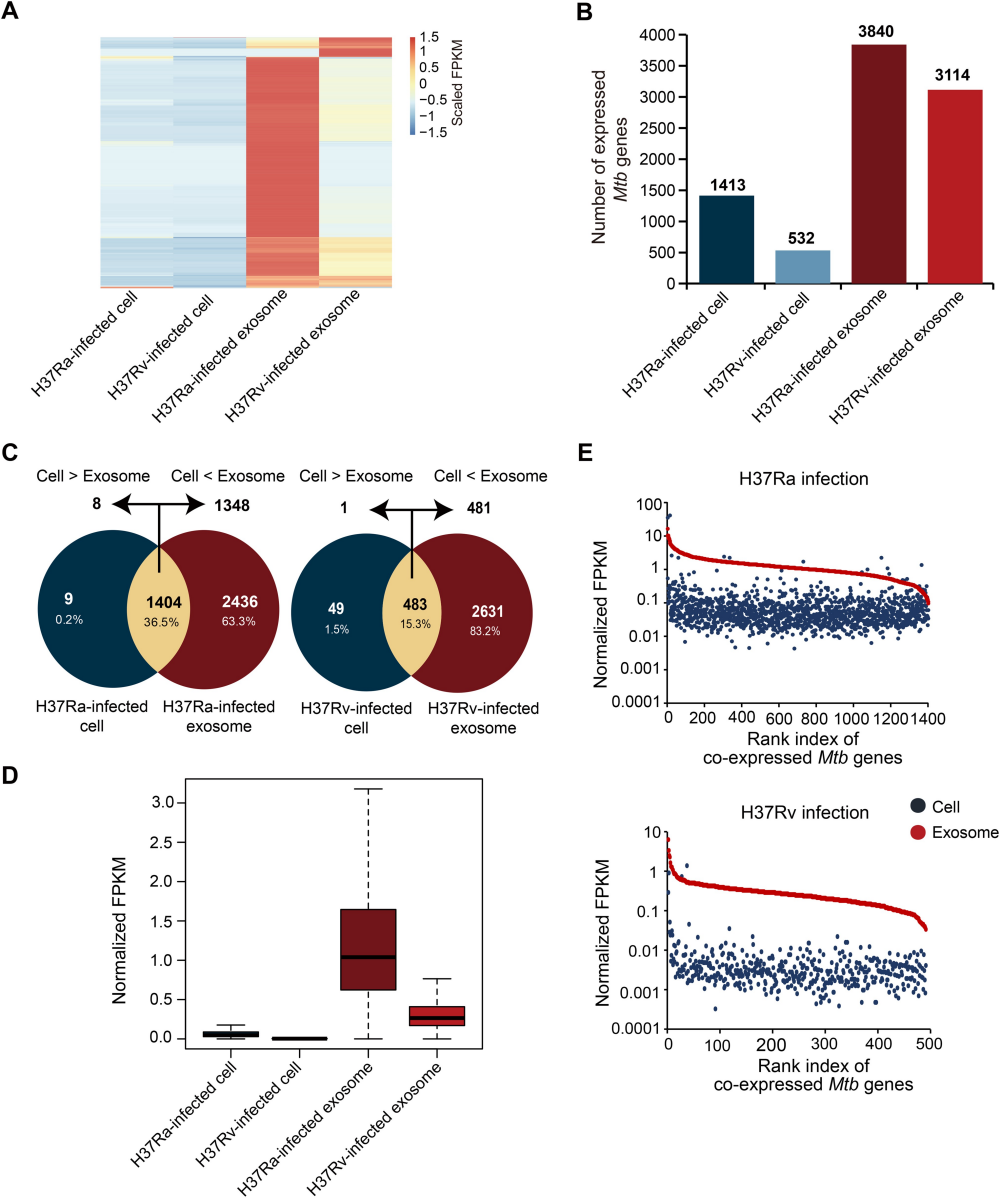
Expression profile of *Mtb* transcripts in the infected macrophages and secreted exosomes **A**. Abundance of *Mtb* transcripts among the infected macrophages and secreted exosomes. **B**. Number of *Mtb* transcripts among the infected macrophages and secreted exosomes. **C**. Venn plots showing the shared expressed *Mtb* genes in the infected cells and exosomes. **D**. Boxplot showing the abundance (normalized FPKM values) of *Mtb* genes in the infected cells and their secreted exosomes. **E**. Abundance of all shared expressed *Mtb* genes (normalized FPKM values) in the infected cells and their secreted exosomes. The red and blue points represent the abundance of genes in exosomes and corresponding macrophages, respectively. Each point represents an *Mtb* gene.

To assess the survival status of *Mtb*, we measured *Mtb* RNA levels in both infected macrophages and their secreted exosomes. As shown in **Figure 6**A, *Mtb* RNAs were significantly enriched in exosomes compared to the corresponding macrophages. Further analysis revealed more *Mtb* genes in exosomes (3114/3840) than in infected macrophages (532/1413) (Figure 6B). Specifically, 2631 (83.2%) and 2436 (63.3%) *Mtb* genes were uniquely detected in H37Rv- and H37Ra-derived exosomes, respectively, with only 49 (1.5%) and 9 (0.2%) identified in the corresponding macrophages (Figure 6C). Moreover, the majority of co-expressed *Mtb* genes had much higher abundance in exosomes than in macrophages: 96.01% (1348/1404) for H37Rv and 99.59% (481/483) for H37Ra infections (Figure 6C and D). The abundance of these co-expressed genes in exosomes was independent of their expression in macrophages (Figure 6E), suggesting directed transport to exosomes.

Notably, H37Ra-derived exosomes contained more expressed genes and higher levels of *Mtb* RNAs compared to H37Rv-derived exosomes (Figure 6A, B, and D). This is consistent with previous findings [12], which suggested that H37Ra is cleared by macrophage apoptosis during robust immune responses, resulting in the secretion of *Mtb* RNAs and other pathogen-associated molecular patterns (PAMPs) in exosomes. In contrast, H37Rv survives within macrophages through immune escape mechanisms.

### High inhibitory effect of treated exosomes and IFN-γ on *Mtb* infection

**Figure 7:**
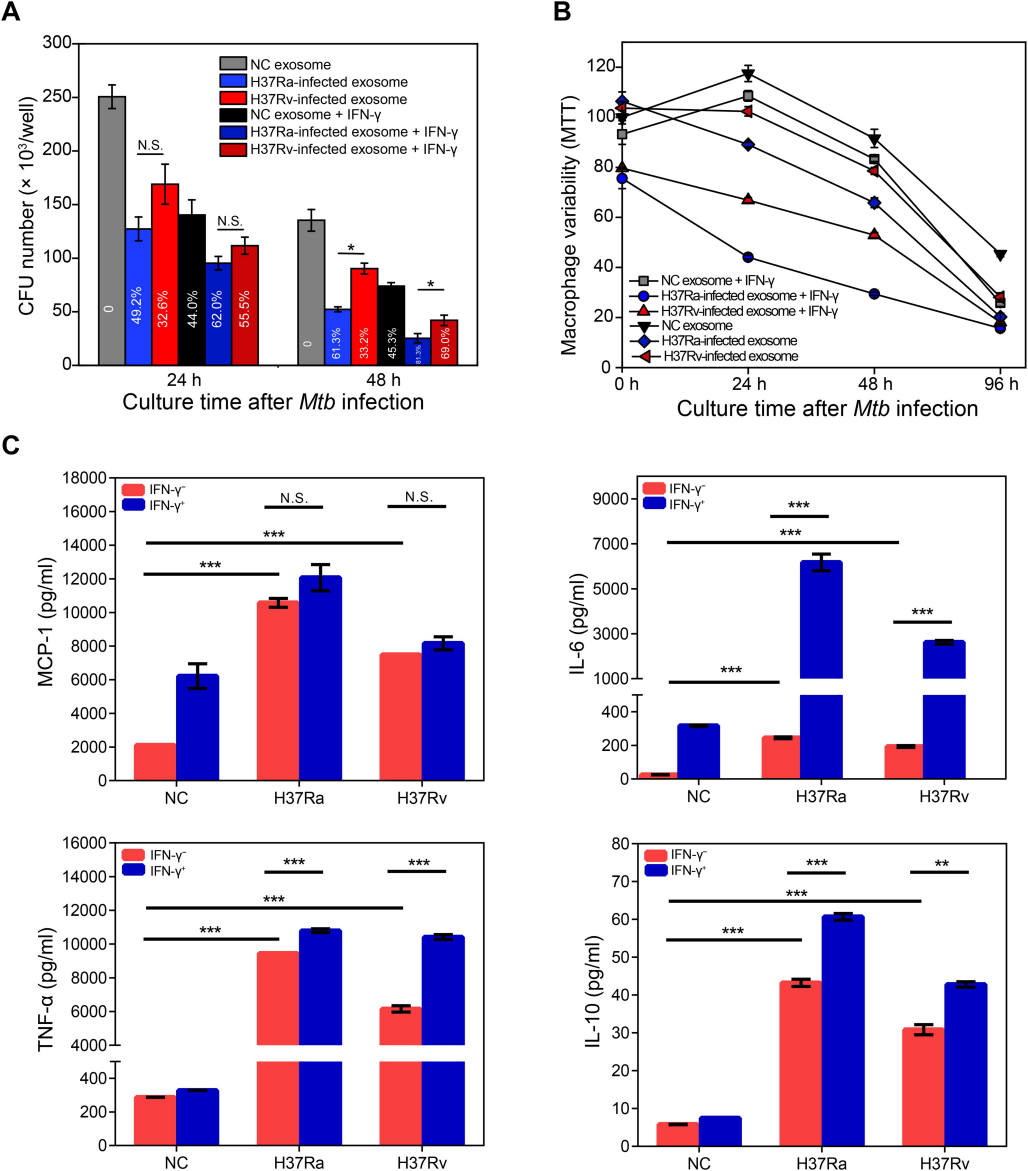
Anti-TB effect of exosomes from *Mtb*-infected macrophages **A**. CFU assay showing the viability of the invading H37Rv in macrophages (MOI = 10) after exosome and/or IFN-γ treatments. The inhibition rate is shown inside each bar. **B**. MTT assay displaying the survival status of infected macrophages (MOI = 10) after exosome and/or IFN-γ treatment. **C**. ELISA demonstrating the expression of TNF-α, IL-6, IL-10, and MCP-1 in the macrophages treated with exosomes and/or IFN-γ. Data are presented as mean ± SEM, and are representative of three independent experiments. All data are from three independent experiments with biological replicates in each (mean ± SEM, *n* = 3 replicates). Statistical significance was determined by two-way ANOVA followed by Tukey’s multiple comparisons test. N.S., not significant; *, *P* < 0.05; **, *P* < 0.01; ***, *P* < 0.001. ELISA, enzyme-linked immunosorbent assay.

To explore the inhibitory effects of treated exosomes and IFN-γ on *Mtb* infection, THP-1 macrophages were treated with exosomes derived from H37Rv- or H37Ra-infected cells, with or without IFN-γ, for 24 h. After treatment, equimolar H37Rv was added, and the growth of cells and invading *Mtb* was measured at 24 h and 48 h using MTT and CFU assays to assess the inhibitory effect (**Figure 7**A and B). The CFU assays revealed that H37Ra-treated exosomes exhibited significantly stronger antibacterial activity than H37Rv-treated exosomes or IFN-γ alone at 48 h (*P* < 0.05). The inhibition rates at 48 h were as follows: 81.3% (H37Ra-exosomes + IFN-γ), 69.0% (H37Rv-exosomes + IFN-γ), 61.3% (H37Ra-exosomes), 33.2% (H37Rv-exosomes), and 45.3% (IFN-γ alone). CFU data at 96 h post-infection were not shown, as negligible colonies were observed, likely due to the poor condition of the macrophages at this time point (Figure 7B), which deprived *Mtb* of nutrients and prevented its survival.

Interestingly, we found a negative correlation between the inhibitory effect (CFU assay) and cellular viability (MTT assay) (Figure 7A and B). Cellular viability decreased in the following order: controls > IFN-γ > H37Rv-exosomes > H37Ra-exosomes > H37Rv-exosomes + IFN-γ > H37Ra-exosomes + IFN-γ. The cell viability of the control and IFN-γ groups initially increased and then decreased [24], whereas all other groups exhibited a continuous decline in cell viability from the onset of infection.

To investigate the mechanism underlying the inhibitory effects of treated exosomes and IFN-γ on H37Rv infection, we measured the expression levels of several key cytokines (IL-6, IL-10, TNF-α, and MCP-1) in macrophages (Figure 7C), as these cytokines are known to play crucial roles in the immune response to *Mtb* infection [31]. As expected, both H37Ra- and H37Rv-treated exosomes promoted cytokine expression, with stronger effects observed with H37Ra-treated exosomes. The addition of exogenous IFN-γ further enhanced cytokine production.

### Differential enrichment of pro-inflammatory and immune escape-related *Mtb* proteins in H37Ra-treated and H37Rv-treated exosomes

To further investigate why H37Ra-treated exosomes exhibited a higher antibacterial effect than H37Rv-treated exosomes, we conducted a comparative proteomic analysis of *Mtb*-derived proteins in the treated exosomes. We identified 488 differentially expressed proteins (DEPs) between H37Ra- and H37Rv-derived exosomes (fold change > 1.2, *P* < 0.05). Notably, 274 proteins (56.15%) were differentially-expressed in H37Ra-treated exosomes, while 214 proteins (43.85%) were differentially-expressed in H37Rv-treated exosomes. Among these, we focused on 179 functionally significant proteins that may contribute to the antibacterial effects, including 65 antigens, 60 virulence factors, 95 membrane proteins, and 7 PE/PPE proteins. More upregulated DEPs were identified in H37Ra-treated exosomes than in H37Rv-treated exosomes (Figure S3). Furthermore, comparative proteomic and transcriptomic analyses revealed only 72 overlapping DEPs/genes, likely due to the distinct mechanisms of RNA and protein secretion (Figure S4).

The top 10 upregulated DEPs in H37Ra-treated exosomes consisted of six membrane proteins, two antigen proteins, one virulence factor, and one multifunctional protein (Table S6). Among these, eight proteins [Hypothetical protein MT1319 (MT1319), Carboxy-terminal protease A (CtpA), Mycobacterial membrane protein Large 8 (Mmpl8), Hypothetical protein MT3772 (MT3772), Mycobacterial membrane protein Small 3 (MmpS3), Ribosomal RNA small subunit methyltransferase A (RsmA), 50S ribosomal protein L35 (RpmI), and Glucose-1-phosphate thymidylyltransferase (RmlA)] are known to be involved in pro-inflammatory responses [32,33]. Additionally, we identified two important immunogenic proteins, Early Secreted Antigenic Target (ESAT)-6-like protein EsxS and the 14 kDa antigen HspX, both of which are known to trigger strong immune responses against *Mtb* and other pathogens [34].

In contrast, the top 10 upregulated DEPs in H37Rv-treated exosomes included five membrane proteins, three antigen proteins, and two virulence factors (Table S6). Importantly, seven of these DEPs [Fumarate reductase subunit D (FrdD), Dormancy survival sensor kinase (DevS), Mycofactocin biosynthesis protein C (MftC), Multicopper oxidase (MmcO), Alcohol dehydrogenase D (AdhD), Hypothetical protein MT3488 (MT3488), and Acetyl-CoA acetyltransferase (FadA)] have been reported to play roles in *Mtb* survival by inhibiting phagosome maturation [35–41], suggesting that H37Rv may escape immune surveillance by selectively secreting pathogenic proteins into exosomes.

## Discussion

### Selective or directed secretion mechanism of host and *Mtb* RNAs from infected macrophages into exosomes

To our knowledge, this is the first comprehensive analysis of the selective secretion and packaging of host and *Mtb* RNAs from infected macrophages into exosomes. For host RNAs, selective packaging is likely mediated by RBPs such as RNA Binding Motif Protein 4 (RBM4), Zinc Finger CCCH-Type Containing 10 (ZC3H10), RNA Binding Motif Protein 8A (RBM8A), Fused in Sarcoma (FUS), Peroxisome Proliferator-Activated Receptor Gamma Coactivator-Related Protein 1 (PPRC1), Serine and Arginine Rich Splicing Factor 1 (SRSF1), and Sterile Alpha Motif Domain Containing 4A (SAMD4A), which recognize specific binding motifs (*e.g.*, GCFCGSS, SSAGCGM, RYGCGCB, CGCGC, SSGCGCS, CRSMSGW, and GCKGGHM). These findings align with previous studies: for example, ribonucleoprotein A2B1 (hnRNPA2b1) has been shown to transport specific microRNAs (miRNAs) into exosomes by binding to GGAC motifs [30]; RBP Y-box Binding Protein 1 (YBX1) has been reported to transfer miR-233 into exosomes [42]; and ELAV Like RNA Binding Protein 1 (ELAVL1) has been implicated in the transport of certain long non-coding RNAs into exosomes [43,44].

Regarding *Mtb* RNAs, their significantly higher abundance in exosomes compared to infected cells (Figure 6A) likely results from directed transport from phagolysosomes in macrophages. After being phagocytosed, bacteria are confined in phagosomes, which fuse with lysosomes to form phagolysosomes, where the bacteria are killed and degraded. The bacterial components, including RNAs, are then selectively packaged into exosomes for secretion [17]. Thus, the phagolysosomal microenvironment plays a critical role in enriching *Mtb* RNAs within exosomes.

### Macrophage vaccination with treated exosomes containing some pro-inflammatory *Mtb* proteins as promising TB subunit vaccine candidates

To understand the inhibitory effect of treated exosomes on *Mtb* infection (Figure 7A and B), we analyzed our proteomic data and identified several pro-inflammatory *Mtb* proteins enriched in the exosomes (Figure S3). Notably, H37Ra-treated exosomes exhibited a stronger antibacterial effect than H37Rv-treated ones, which may be attributed to the selective enrichment of immunogenic proteins in the former. Among the top 10 significantly upregulated proteins in H37Ra-treated exosomes (Table S6), eight are known to play roles in inducing pro-inflammatory responses against *Mtb* infection: MmpS3 (a candidate subunit vaccine), Mmpl8 (a promising vaccine component with significant protective effects in a murine TB model), MT1319 (an immune-reactive antigen), CtpA (induces innate immune responses in alveolar macrophages), RsmA (blocks transcription initiation of Esx secreted proteins to eliminate *Mtb*), RpmI (induces dendritic cell maturation and pro-inflammatory cytokine production), and RmlA (involved in PIM and induces inflammation against *Mtb*). These proteins represent promising candidates for TB subunit vaccines, and their therapeutic potential warrants further investigation.

Additionally, several *Mtb* proteins have been identified as exosomal antigens in the fight against *Mtb* infection. For example, a 19 kDa lipoprotein isolated from over 40 exosomal *Mtb* proteins in bronchoalveolar lavage (BAL) fluid of TB patients has been shown to induce pro-inflammatory responses and recruit macrophages and neutrophils [19].

Our findings also reveal a significant enrichment of immune escape-related proteins in H37Rv-treated exosomes (Figure 4; Table S6), which are implicated in inhibiting phagosome maturation and promoting *Mtb* pathogenicity [36,39,45]. Among the top 10 upregulated proteins in H37Rv-treated exosomes, seven are known to support *Mtb* survival: FrdD promotes *Mtb* survival *in vivo*; DevS, part of the devR–devS system, aids *Mtb* pathogenicity and survival; MftC is crucial for mycofactocin synthesis, enhancing *Mtb* niche survival; MmcO protects *Mtb* from ROS; AdhD is involved in lipid biosynthesis for the cell envelope; MT3488, a diterpene synthase, ensures survival inside macrophages by inhibiting phagolysosome maturation and phagocytosis; and FadA supports *Mtb* survival, reactivation, and transmission through lipid droplet production. These immune escape and survival-related proteins highlight how *Mtb* evades host immunity, and targeting them could offer new therapeutic strategies against *Mtb* infection.

Overall, the significant upregulation of immune escape and survival-related proteins in H37Rv-treated exosomes underscores *Mtb*’s ability to evade host immunity. Targeting these proteins could disrupt immune escape mechanisms and provide new therapeutic strategies against *Mtb* infection.

### Compensatory activation of IFN signaling pathways in macrophages in the absence of IFN-γ expression

Interestingly, IFN-γ was predicted as the top upstream regulator in both H37Ra- and H37Rv-infected macrophages, and the “IFN signaling” pathway was highly activated. This is inconsistent with the traditional understanding of IFN-γ’s role during *Mtb* infection [29]. Typically, IFN-γ is produced by CD4^+^ T cells and NK cells, not by macrophages, and plays a crucial role in activating macrophages to combat *Mtb* infection. *In vivo*, the production of IFN-γ and other key antibacterial cytokines is essential for the anti-*Mtb* immune response, involving processes like PAMP recognition, antigen presentation, CD4^+^ T cell activation, and cytokine production [9,29]. Therefore, the significant activation of IFN-γ signaling pathways in macrophages, despite the absence of IFN-γ expression, is both surprising and intriguing.

Both previous studies and our transcriptomic data confirmed that IFN-γ was not produced in the infected macrophages (Table S4) [29]. Nevertheless, IFN signaling pathways were still significantly activated in our study (Figures 3G, 4A and B). This suggests a potential mechanism: other upstream regulators may upregulate downstream genes, thereby compensatorily activating IFN signaling pathways and enabling macrophages to resist *Mtb* infection in an IFN-γ-independent manner (Figure 4C and D). This implies that macrophages may employ a compensatory mechanism to eliminate *Mtb* without IFN-γ. Further investigation is needed to confirm whether this compensatory strategy occurs *in vivo*. Additionally, the activation of IFN signaling pathways in the absence of IFN-γ expression (Figure 4) supports the idea that these pathways are crucial for macrophages to effectively combat invading *Mtb*, and all roads lead to Rome.

### Important role of the HMGB1 signaling pathway in immune escape of virulent *Mtb* for *in vivo* survival

Our study reveals that H37Ra induces a stronger macrophage immune response than H37Rv, both in terms of phenotype (Figure 2B and C) and genotype (Figure 3), consistent with a previous study on cytokine production in H37Ra- and H37Rv-infected macrophages [46]. Notably, the “HMGB1 signaling” pathway was the only immune-related pathway significantly activated in H37Rv-infected macrophages among the 10 activated immune pathways (Figure 4G). HMGB1 has been shown to play a critical role in immune escape, facilitating the *in vivo* survival of *Mtb* by inducing necrosis [47]. Another study also suggested that the HMGB1 pathway contributes to tumor immune escape by increasing myeloid-derived suppressor cells and promoting IL-10 production [25].

Additionally, among the four significantly upregulated genes in the “HMGB1 signaling” pathway, *TNFRSF1B* has been implicated in inhibiting apoptosis of infected macrophages, further aiding the immune escape of *Mtb* [48]. Taken together, these findings suggest that the weaker immune response observed in H37Rv-infected macrophages may be attributed to the activation of the “HMGB1 signaling” pathway, which regulates cell death pathways (blocking apoptosis and promoting necrosis) to facilitate immune evasion and intracellular survival of virulent *Mtb*. Inhibitors targeting the “HMGB1 signaling” pathway, as well as *TNFRSF1B*, may offer promising therapeutic strategies for *Mtb* infection. Further studies are needed to explore this potential.

### Limitation

We utilized an *in vitro* THP-1 cell model, which may not fully represent the complexity of the human immune microenvironment. Due to the revocation of our hospital’s license for handling *Mtb* infections in animals (Beijing Chest Hospital) as part of Beijing’s unified planning, we were unable to perform validation experiments on the promising candidates identified in this study. Despite these limitations, our research underscores the complexity of macrophage immune responses to *Mtb* infection, highlighting the need for further studies to fully elucidate the underlying mechanisms.

## Conclusion

Macrophages play a pivotal role in the immune response against *Mtb*. However, a comprehensive understanding of the immune mechanisms in infected macrophages remains lacking. In this study, we conducted detailed analyses of macrophages infected with avirulent (H37Ra) and virulent (H37Rv) *Mtb* and addressed four key points: (1) avirulent *Mtb* stimulates robust immune responses and apoptosis to eliminate the bacteria, while virulent *Mtb* induces necrosis and immune evasion for intracellular survival; (2) macrophages kill *Mtb* in an IFN-γ-independent manner, emphasizing the importance of the IFN signaling pathway in the anti-TB immune response; (3) we identified selective secretion and transport of both host and *Mtb* RNAs into exosomes; and (4) *Mtb*-treated exosomes exhibit strong anti-TB effects, likely due to the presence of pro-inflammatory/immunogenic *Mtb* proteins. Overall, our findings provide new insights into macrophage immune mechanisms against *Mtb* infection and suggest novel TB vaccine candidates.

## Materials and methods

### Cell line and culture

Human THP-1 cells were cultured in 10 ml Roswell Park Memorial Institute (RPMI) 1640 medium (Catalog No. FG1385, Biochrom, Berlin, Germany) with 10% (v/v) Fetal Bovine Serum (FBS; Catalog No. 10099141C, GIBCO BRL, Grand Island, NY), 1 mM *L*-glutamine, 100 U/ml penicillin, and 100 µg/ml streptomycin (Catalog No. 85886, Sigma-Aldrich, St. Louis, MO). THP-1 monocytes were differentiated into macrophages by incubation with 50 nM PMA (Catalog No. P8139, Sigma-Aldrich) for 24 h.

### Bacterial strains and culture

Virulent and avirulent *Mtb* reference strains H37Rv and H37Ra were cultured at 37°C in Middlebrook 7H9 medium (Catalog No. 271310, BD Biosciences, San Jose, CA) supplemented with 10% (v/v) oleic acid albumin dextrose catalase (OADC; Catalog No. M0678, Sigma-Aldrich), 0.5% glycerol, and 0.1% Tween-80 or on 7H10 plates (Catalog No. 271320, BD Biosciences) with 10% OADC and 0.5% glycerol.

### Cell infection and *Mtb* staining

*Mtb* strains were inoculated into 7H9 medium and cultured to an Optical Density (OD) = 1.0. The differentiated THP-1 macrophage cells (2 × 10^7^) were infected with *Mtb* at a multiplicity of infection (MOI) of 10. After 4 h of phagocytosis, infected cells were washed with Phosphate Buffered Saline (PBS) before being replaced with exosome-free RPMI 1640 medium. Cells and supernatant were collected after 24 h post-infection. Intracellular bacteria were identified using the ZN stain according to the manufacturer’s instructions and visualized under a microscope.

### Exosome extraction

Exosomes from *Mtb*-infected THP-1 macrophages were isolated as previously described [22,23]. Transmission electron microscopy (TEM; Catalog No. JEM-1400, JEOL, Tokyo, Japan) was used to visualize the exosomes. The size distribution of exosomes was measured by NTA (Catalog No. NS300, Malvern, UK). Additionally, TEM combined with immunogold labeling (for surface markers CD9 and CD81) was used to visualize exosomes, with calnexin, an endoplasmic reticulum marker protein, serving as a negative control (Figure S5).

### CFU and MTT assay

The infected macrophages were washed with PBS, lysed in 0.5% Triton-X at 37°C, serially diluted in PBS with 0.05% Tween-80, plated onto 7H10 plates, and counted for CFU after three weeks.

The infected macrophages were incubated with 10 μl MTT reagent at 8 h and 24 h post-infection in a cell incubator with 5% CO_2_ at 37°C for 4 h. The culture medium was then removed, and 100 μl Dimethyl Sulfoxide (DMSO) was added to each well. The absorbance was measured at OD_590_ using Enzyme Linked Immunosorbent Assay (ELISA) Reader (EnSpire^®^ Multimode Plate Reader, EnSpire, Waltham, MA).

### Cell treatment with exosomes and/or IFN-γ

Differentiated THP-1 macrophages were treated with the extracted exosomes at a ratio (exosome:cell) of 100:1, and/or IFN-γ (1000 U/ml) for 24 h before infection.

### Cytokine detection

Human TNF-α, IL-6, MCP-1, and IL-10 from the treated THP-1 macrophages were detected using MILLIPLEX^®^ milliplex assay (HCYTOMAG-60K-08, Millipore, Burlington, MA), and analyzed using a Luminex 200 platform.

### Liquid chromatography-tandem mass spectrometry analysis using isobaric tags for relative and absolute quantitation

Exosomes were lysed in lysis buffer with protease inhibitors, and protein concentrations were measured using a BCA Protein Assay Kit (Catalog No. 23225, Thermo Scientific Pierce, Waltham, MA). Samples (100 µg) from H37Ra- and H37Rv-treated exosomes were labelled with 8-plex iTRAQ reagents, acidified, mixed, and desalted with C18 tips, as previously reported [49]. Eluted samples were dried by SpeedVac for mass spectrometry (MS) analysis [50].

Liquid chromatography-tandem MS** (**LC-MS/MS) analysis was run on TripleTOF 6600 MS (AB Sciex) equipped with an Eksigent ekspert nanoLC 425 system [51,52]. Peptides were loaded onto a Nano Trap column and separated on an analytical column using a linear 65-min gradient of buffer B at a flow rate of 300 nl/min. MS data were acquired in positive ion mode using a data-dependent top 10 method, with a dynamic exclusion duration of 40 s. Resulting spectra were analyzed using Proteome Discoverer (PD) software with the Mascot search engine. The 1.2-fold change was used as a cut-off for biological significance based on normalized protein ratios.

### RNA sequencing, data processing, and bioinformatic analysis

Total RNAs of the *Mtb*-infected cells and isolated exosomes were extracted using RNAiso Plus (Catalog No. 9109, Takara, Tokyo, Japan) according to the manufacturer’s instructions. RNA concentration was measured using Qubit^®^ RNA Assay Kit in Qubit^®^ 2.0 Fluorometer (Catalog No. Q32866, Invitrogen, Carlsbad, CA). RNA integrity was assessed using the RNA 6000 Nano Assay Kit of the Agilent Bioanalyzer 2100 system (Agilent Technologies, Santa Clara, CA).

Sequencing libraries were constructed using NEBNext^®^ Ultra^TM^ Directional RNA Library Prep Kit for Illumina^®^ (Catalog No. E7420S, New England Biolabs, Ipswich, MA). The library quality was assessed on the Agilent Bioanalyzer 2100 system. The RNA libraries were sequenced on the Illumina HiSeq 2500 Genome Analyzer platform (HiSeq 2500, Illumina, San Diego, CA) in paired-end mode. Raw RNA sequencing (RNA-seq) reads were filtered to obtain the clean RNA reads (FastQC) for subsequent analysis.

The clean data for each sample were mapped to the human reference genome GRCh38 using TopHat (v2.1.0). The unique mapping reads were spliced and assembled using Cufflinks (v2.2.1), and transcripts from the same gene were normalized to obtain the expression. DEGs among samples were obtained using Cuffdiff, with the following selection criteria: *P* < 0.05 and absolute fold change > 2.

To detect the *Mtb* transcripts, the clean data were mapped to the genomes of H37Ra and H37Rv using the Burrows–Wheeler Alignment (BWA) tool. The expression levels of *Mtb* genes in cells and exosomes were determined using Cufflinks.

Bioinformatic analyses were conducted using IPA software, which integrates knowledge curated by over 200 PhD experts. This tool supports functional analysis, pathway enrichment, and upstream regulator prediction by assessing gene expression changes and considering the directionality of these changes based on curated biological evidence. The analysis is quantified using a Z-score, with an absolute Z-score > 2 deemed significant. A higher Z-score indicates stronger activation, while a lower value suggests inhibition. For more details on IPA and the Z-score, please refer to Krämer and colleagues [53]. IPA’s upstream regulator analysis is a significant advancement in predicting transcriptional regulators. Unlike other tools, IPA predicts which transcriptional regulators are involved and whether they are likely to be activated or inhibited, based on curated evidence. It also visualizes the network of regulators and their targets, revealing their interactions and offering testable hypotheses for gene regulatory networks.

## Code availability

The code for comparative transcriptomic analysis of macrophages infected with avirulent and virulent *Mtb* strains has been submitted to BioCode at the National Genomics Data Center (NGDC), China National Center for Bioinformation (CNCB) (BioCode: BT007697), which is publicly accessible at https://ngdc.cncb.ac.cn/biocode/tool/BT7697.

## Supplementary Material

qzaf065_Supplementary_Data

## Data Availability

The raw sequencing data of this study have been deposited in the Genome Sequence Archive for Human [54] at the NGDC, CNCB [55] (GSA-human: HRA004324), which are publicly accessible at https://ngdc.cncb.ac.cn/gsa-human. The mass spectrometry proteomics data of exosomes from Mtb-infected macrophages have been deposited in the ProteomeXchange Consortium via the iProX partner repository (ProteomeXchange: PXD078478).

## CRediT author statement

**Li Yang:** Validation, Formal analysis, Writing – original draft, Resources, Visualization. **Lingna Lyu:** Validation, Resources. **Cuidan Li:** Formal analysis, Visualization, Writing – review & editing. **Xiuli Zhang:** Validation, Resources. **Yingjiao Ju:** Formal analysis, Writing – original draft. **Ju Zhang:** Conceptualization, Resources. **Jie Liu:** Formal analysis, Resources. **Liya Yue:** Validation, Resources. **Nan Ding:** Visualization. **Xiangli Zhang:** Formal analysis, Resources. **Dandan Lu:** Formal analysis, Resources. **Tingting Yang:** Formal analysis. **Peihan Wang:** Resources. **Jie Wang:** Visualization. **Xiaotong Wang:** Visualization; **Sihong Xu:** Visualization. **Yongjie Sheng:** Visualization. **Chunlai Jiang:** Resources. **Jing Wang:** Visualization. **Xin Hu:** Visualization. **Bahetibieke Tuohetaerbaike** Visualization. **Zongde Zhang:** Conceptualization, Writing – review & editing, Resources, Supervision. **Fei Chen:** Conceptualization, Writing – review & editing, Funding acquisition, Project administration, Supervision. All authors have read and approved the final manuscript.

## ORCID

0000-0001-7353-4415 (Li Yang)

0000-0002-4154-8094 (Lingna Lyu)

0000-0002-8430-1155 (Cuidan Li)

0000-0002-5694-0101 (Xiuli Zhang)

0000-0002-3842-850X (Yingjiao Ju)

0000-0003-3274-9844 (Ju Zhang)

0009-0008-1167-1674 (Jie Liu)

0000-0002-5752-6914 (Liya Yue)

0000-0002-1045-1695 (Nan Ding)

0000-0003-4816-6598 (Xiangli Zhang)

0000-0001-6977-4301 (Dandan Lu)

0000-0002-8585-0922 (Tingting Yang)

0000-0001-6701-6179 (Peihan Wang)

0000-0003-3366-2783 (Jie Wang)

0000-0002-9867-418X (Xiaotong Wang)

0000-0002-1899-0781 (Sihong Xu)

0000-0002-4402-0500 (Yongjie Sheng)

0000-0002-4497-5315 (Chunlai Jiang)

0000-0003-3318-2130 (Jing Wang)

0009-0002-6922-8372 (Xin Hu)

0000-0003-1544-9380 (Bahetibieke Tuohetaerbaike)

0000-0003-0857-9793 (Zongde Zhang)

0000-0003-3895-6318 (Fei Chen)
