# Supplementary material for: Macrophage Response to Avirulent and Virulent Mycobacterium tuberculosis and Anti-TB Effects of Exosome Treatment
Source: Genomics Proteomics Bioinformatics. 2025 Aug 5;23(6):qzaf065. doi: 10.1093/gpbjnl/qzaf065 (PMC13234453; doi:10.1093/gpbjnl/qzaf065)
Supplement: qzaf065_Supplementary_Data [file qzaf065_supplementary_data.zip › supplementary material captions.docx]

**Supplementary materials**

**Figure S1 RNA sequencing of H37Ra-infected and H37Rv-infected macrophages and released exosomes**

**A.** Cellular and exosomal RNAs are assessed on the Agilent Bioanalyzer 2100 system (Agilent Technologies, CA, USA). Typical 28S and 18S rRNA peaks are detected in the cellular RNAs. Exosomal RNAs are mainly distributed between 22–32 nt. **B.** Pointplot showing the correlation between two independent repeats. **C.** Saturation curves of sequencing data. rRNA, ribosomal RNA; FU, Fluorescent unit.

**Figure S2 Bubble plot showing the enriched GO terms for the genes with above-mentioned eight motifs**

The color indicates the adjust *P* value; the size of dots indicates the gene ratio. GO, Gene Ontology.

**Figure S3 Bar plot representing 179 important functional DEPs in the H37Ra-treated and H37Rv-treated exosomes**

The blue and orange bars represent the upregulated *Mtb* DEPs in the H37Ra-treated and H37Rv-treated exosomes, respectively (H37Ra/H37Rv ratio > 1.2, *P* value < 0.05). DEP, differently expressed protein.

**Figure S4 Venn diagram illustrating the overlap between transcriptomic and proteomic results**

DEGs represent differentially expressed genes and DEPs represent differentially expressed proteins.

**Figure S5 Identification of exosomes by TEM combined with immunogold labeling**

Exosome surface marker CD9 and CD81 were detected with rabbit anti-human antibodies of anti-CD9 and anti-CD81 (EXOAB-CD9A-1, EXOAB-CD81A-1, SBI, CA, USA), and an endoplasmic reticulum marker protein, calnexin, as negative control was detected with anti-calreticulin antibody (ab2907, Abcam, MA, USA).

**Table S1 Comparison of upregulated DEGs related to apoptosis and immune escape between H37Ra and H37Rv**

**Table S2 The top 25 predicted upstream regulators for all DEGs in the H37Ra infected macrophages**

**Table S3 The top 25 predicted upstream regulators for all DEGs in the H37Rv infected macrophages**

**Table S4 Expression of type I and II interferons in the H37Ra-infected and H37Rv-infected macrophages**

**Table S5 Specifically enriched RBP recognition motifs in the exosomes derived from H37Ra and H37Rv infections**

**Table S6 The top 10 significantly upregulated differentially expressed functional proteins in the H37Ra-treated and H37Rv-treated exosomes**
