# Supplementary material for: Macrophage Response to Avirulent and Virulent Mycobacterium tuberculosis and Anti-TB Effects of Exosome Treatment
Source: Genomics Proteomics Bioinformatics. 2025 Aug 5;23(6):qzaf065. doi: 10.1093/gpbjnl/qzaf065 (PMC13234453; doi:10.1093/gpbjnl/qzaf065)
Supplement: qzaf065_Supplementary_Data [file qzaf065_supplementary_data.zip › Table S1.docx]

**Table S1 Comparison of upregulated DEGs related to apoptosis and immune escape between H37Ra and H37Rv**

| **Treatment** | **Upregulated DEGs number** | **Apoptosis-related DEGs number** | **Immune escape-related DEGs number** |
| --- | --- | --- | --- |
| H37Ra | 181 | 16 | 4 |
| H37Rv | 158 | 9 | 9 |
