# Supplementary material for: Macrophage Response to Avirulent and Virulent Mycobacterium tuberculosis and Anti-TB Effects of Exosome Treatment
Source: Genomics Proteomics Bioinformatics. 2025 Aug 5;23(6):qzaf065. doi: 10.1093/gpbjnl/qzaf065 (PMC13234453; doi:10.1093/gpbjnl/qzaf065)
Supplement: qzaf065_Supplementary_Data [file qzaf065_supplementary_data.zip › Table S2.docx]

**Table S2**  **The top 25 predicted upstream regulators for all DEGs in the H37Ra infected macrophages**

| **Upstream regulator** | **Type** | **Z-score** | ***P* value** |
| --- | --- | --- | --- |
| IFNG | Cytokine | 6.064 | 7.53E−27 |
| poly rI:rC-RNA | Biologic drug | 5.752 | 1.40E−32 |
| Interferon alpha | Ggroup | 5.244 | 2.67E−27 |
| Ifnar | Group | 5.078 | 7.98E−31 |
| STAT1 | Transcription regulator | 4.869 | 9.98E−28 |
| TLR3 | Transmembrane receptor | 4.712 | 3.25E−31 |
| TNF | Cytokine | 4.687 | 1.78E−18 |
| TLR9 | Transmembrane receptor | 4.58 | 1.48E−22 |
| IRF7 | Transcription regulator | 4.52 | 4.21E−23 |
| MYD88 | Other | 4.454 | 8.08E−17 |
| IRF3 | Transcription regulator | 4.342 | 6.48E−33 |
| IRF5 | Transcription regulator | 4.323 | 2.90E−26 |
| IFNB1 | Cytokine | 4.307 | 3.44E−26 |
| TICAM1 | Other | 4.253 | 1.20E−20 |
| IL1B | Cytokine | 4.171 | 5.57E−14 |
| TLR4 | Transmembrane receptor | 4.121 | 1.36E−20 |
| Ifn | Group | 4.028 | 9.15E−21 |
| IL21 | Cytokine | 3.934 | 1.90E−12 |
| CHUK | Kinase | 3.912 | 2.18E−14 |
| SAMSN1 | Other | 3.9 | 1.75E−18 |
| IKBKB | Kinase | 3.653 | 2.40E−10 |
| ACKR2 | G-protein coupled receptor | −3.742 | 5.25E−18 |
| PTGER4 | G-protein coupled receptor | −3.889 | 7.87E−19 |
| IL10RA | Transmembrane receptor | −4.359 | 1.16E−09 |
| TRIM24 | Transcription regulator | −4.67 | 3.50E−22 |
