# Supplementary material for: Macrophage Response to Avirulent and Virulent Mycobacterium tuberculosis and Anti-TB Effects of Exosome Treatment
Source: Genomics Proteomics Bioinformatics. 2025 Aug 5;23(6):qzaf065. doi: 10.1093/gpbjnl/qzaf065 (PMC13234453; doi:10.1093/gpbjnl/qzaf065)
Supplement: qzaf065_Supplementary_Data [file qzaf065_supplementary_data.zip › Table S3.docx]

**Table S3 The top 25 predicted upstream regulators for all DEGs in the H37Rv infected macrophages**

| **Upstream regulator** | **Type** | **Z-score** | ***P* value** |
| --- | --- | --- | --- |
| IFNG | Cytokine | 5.698 | 3.33E−26 |
| poly rI:rC-RNA | Biologic drug | 5.592 | 1.73E−29 |
| IRF3 | Transcription regulator | 5.443 | 1.20E−37 |
| Ifnar | Group | 5.280 | 5.47E−37 |
| STAT1 | Transcription regulator | 5.054 | 1.40E−29 |
| IRF7 | Transcription regulator | 5.028 | 1.01E−33 |
| Interferon alpha | Group | 4.758 | 3.14E−23 |
| IRF5 | Transcription regulator | 4.463 | 3.76E−30 |
| TLR3 | Transmembrane receptor | 4.442 | 8.24E−21 |
| TICAM1 | Other | 4.265 | 2.06E−17 |
| IFNB1 | Cytokine | 4.256 | 2.52E−25 |
| NFATC2 | Transcription regulator | 4.176 | 6.14E−16 |
| TLR9 | Transmembrane receptor | 4.031 | 6.67E−14 |
| MYD88 | Other | 3.946 | 1.23E−10 |
| Ifn | Group | 3.903 | 2.19E−18 |
| SAMSN1 | Other | 3.873 | 1.23E−14 |
| IL21 | Cytokine | 3.873 | 1.06E−12 |
| TNF | Cytokine | 3.846 | 4.93E−07 |
| TLR4 | Transmembrane receptor | 3.581 | 4.45E−13 |
| TMEM173 | Other | 3.440 | 4.94E−17 |
| NFE2L2 | Transcription regulator | −3.120 | 4.06E−03 |
| ACKR2 | G-protein coupled receptor | −4.243 | 4.65E−27 |
| IL10RA | Transmembrane receptor | −4.243 | 1.81E−10 |
| PTGER4 | G-protein coupled receptor | −4.408 | 2.99E−18 |
| TRIM24 | Transcription regulator | −5.177 | 2.53E−32 |
