# Supplementary material for: Macrophage Response to Avirulent and Virulent Mycobacterium tuberculosis and Anti-TB Effects of Exosome Treatment
Source: Genomics Proteomics Bioinformatics. 2025 Aug 5;23(6):qzaf065. doi: 10.1093/gpbjnl/qzaf065 (PMC13234453; doi:10.1093/gpbjnl/qzaf065)
Supplement: qzaf065_Supplementary_Data [file qzaf065_supplementary_data.zip › Table S4.docx]

**Table S4 Expression of type I and II interferons in the H37Ra-infected and H37Rv-infected macrophages**

| **Gene name** | **Gene ID** | **FPKM (H37Ra)** | **FPKM (H37Rv)** |
| --- | --- | --- | --- |
| *IFNG* | ENSG00000111537.4 | 0 | 0 |
| *IFNA6* | ENSG00000120235.4 | 0 | 0 |
| *IFNA8* | ENSG00000120242.3 | 0 | 0 |
| *IFNA21* | ENSG00000137080.4 | 0 | 0 |
| *IFNA5* | ENSG00000147873.5 | 0 | 0 |
| *IFNA16* | ENSG00000147885.4 | 0 | 0 |
| *IFNK* | ENSG00000147896.3 | 0 | 0 |
| *IFNB1* | ENSG00000171855.6 | 0.953749 | 0.390445 |
| *IFNW1* | ENSG00000177047.6 | 0 | 0 |
| *IFNL1* | ENSG00000182393.2 | 0.0775645 | 0.0736963 |
| *IFNL2* | ENSG00000183709.7 | 0 | 0 |
| *IFNE* | ENSG00000184995.7 | 0 | 0 |
| *IFNA10* | ENSG00000186803.3 | 0 | 0 |
| *IFNA2* | ENSG00000188379.6 | 0 | 0 |
| *IFNL3* | ENSG00000197110.8 | 0 | 0 |
| *IFNA1* | ENSG00000197919.4 | 0 | 0 |
| *IFNA7* | ENSG00000214042.1 | 0 | 0 |
| *IFNWP18* | ENSG00000223684.1 | 0 | 0 |
| *IFNA22P* | ENSG00000224416.2 | 0 | 0 |
| *IFNWP4* | ENSG00000225027.2 | 0 | 0 |
| *IFNA20P* | ENSG00000226393.1 | 0 | 0 |
| *IFNWP9* | ENSG00000226597.1 | 0 | 0 |
| *IFNA14* | ENSG00000228083.2 | 0 | 0 |
| *IFNNP1* | ENSG00000230208.1 | 0 | 0 |
| *IFNA11P* | ENSG00000231195.1 | 0 | 0 |
| *IFNWP5* | ENSG00000232138.1 | 0 | 0 |
| *IFNWP15* | ENSG00000232281.2 | 0 | 0 |
| *IFNA13* | ENSG00000233816.3 | 0 | 0 |
| *IFNA17* | ENSG00000234829.3 | 0 | 0 |
| *IFNA12P* | ENSG00000235108.1 | 0 | 0 |
| *IFNA4* | ENSG00000236637.2 | 0 | 0.040364 |
| *IFNWP2* | ENSG00000237691.1 | 0 | 0 |
| *IFNWP19* | ENSG00000238271.2 | 0 | 0 |
| *IFNG-AS1* | ENSG00000255733.5 | 0.0814241 | 0 |
| *IFNL3P1* | ENSG00000268510.1 | 0 | 0 |
| *IFNL4P1* | ENSG00000272311.1 | 0 | 0 |
| *IFNL4* | ENSG00000272395.5 | 0 | 0 |
