# Supplementary material for: Macrophage Response to Avirulent and Virulent Mycobacterium tuberculosis and Anti-TB Effects of Exosome Treatment
Source: Genomics Proteomics Bioinformatics. 2025 Aug 5;23(6):qzaf065. doi: 10.1093/gpbjnl/qzaf065 (PMC13234453; doi:10.1093/gpbjnl/qzaf065)
Supplement: qzaf065_Supplementary_Data [file qzaf065_supplementary_data.zip › Table S5.docx]

**Table S5**  **Specifically enriched RBP recognition motifs in the exosomes derived from H37Ra and H37Rv infections**

| **Motif** | **RNA-binding protein** | **Enrichment** | **Function** |
| --- | --- | --- | --- |
| gcgcgss | RBM4 | Ra & Rv | Alternative splicing of pre-mRNA and translation regulation |
| ssagcgm | ZC3H10 | Ra & Rv | miRNA binding; mitochondrial physiology regulator |
| rygcgcb | RBM8A | Ra & Rv | Spliced mRNAs |
| cgcgc | FUS | Ra & Rv | RNA transporting; pre-mRNA splicing and the export of fully processed mRNA to the cytoplasm |
| ssgcgcs | PPRC1 | Ra & Rv | Transcription factor binding; nuclear receptor transcription coactivator activity |
| crsmsgw | SRSF1 | Ra & Rv | Regulating alternative splicing |
| gckgghm | SAMD4A | Ra & Rv | mRNA binding; translation repressor activity |
| dgacrrr | FXR2 | Ra only | RNA binding; translational control and RNA transport. |

*Note*: Ra represents H37Ra infection group; Rv represents H37Rv infection group. RBP, RNA-binding protein; pre-mRNA, precursor messenger RNA; mRNA, messenger RNA; miRNA, microRNA.
