# Supplementary material for: Macrophage Response to Avirulent and Virulent Mycobacterium tuberculosis and Anti-TB Effects of Exosome Treatment
Source: Genomics Proteomics Bioinformatics. 2025 Aug 5;23(6):qzaf065. doi: 10.1093/gpbjnl/qzaf065 (PMC13234453; doi:10.1093/gpbjnl/qzaf065)
Supplement: qzaf065_Supplementary_Data [file qzaf065_supplementary_data.zip › Table S6.docx]

| **Table S6**  **The top 10 significantly upregulated differentially expressed functional proteins in the H37Ra-infected and H37Rv-treated exosomes** | | | | | | | | | |
| --- | --- | --- | --- | --- | --- | --- | --- | --- | --- |
| **Gene ID** | **Protein name** | **Ranking** | **Rv-Exo abundance** | **Ra-Exo abundance** | **Ra-Exo/Rv-Exo ratio** | **VF gene** | **Antigen** | **Membrane** | **Reference** |
| Rv2198c | MmpS3 | 1 | 27.6 | 172.4 | 6.25 |  |  | Y | PMID: 31652116 |
| Rv3219 | WhiB1 | 2 | 27.8 | 172.2 | 6.19 |  | Y |  | PMID: 24891105 |
| Rv1282c | MT1319 | 3 | 34 | 166 | 4.88 |  |  | Y | PMID: 18039835 |
| Rv0092 | CtpA | 4 | 39 | 161 | 4.13 |  |  | Y | PMID: 25703564 |
| Rv3823c | Mmpl8 | 5 | 42.8 | 157.2 | 3.67 | Y | Y | Y | PMID: 29618733 |
| Rv3912 | RsmA | 6 | 43.1 | 156.9 | 3.64 |  |  | Y | PMID: 19951358 |
| Rv2578c | MT2655 | 7 | 43.7 | 156.3 | 3.58 |  |  | Y | PMID: 18083815 |
| Rv1642 | RpmI | 8 | 45.6 | 154.4 | 3.39 |  | Y |  | PMID: 25102137 |
| Rv0334 | RmlA | 9 | 49.2 | 150.8 | 3.07 | Y |  |  | PMID: 31728063 |
| Rv3671c | MT3772 | 10 | 49.4 | 150.6 | 3.05 |  |  | Y | PMID: 30366125 |
| Rv0859 | FadA | 10 | 155.7 | 44.3 | 0.28 |  |  | Y | PMID: 30644849 |
| Rv3378c | MT3488 | 9 | 157.1 | 42.9 | 0.27 |  | Y |  | PMID: 24475925 |
| Rv3086 | AdhD | 8 | 158.2 | 41.8 | 0.26 | Y |  |  | PMID: 11257547 |
| Rv0846c | MmcO | 7 | 158.6 | 41.4 | 0.26 |  |  | Y | PMID: 31278055 |
| Rv0693 | MftC | 6 | 159 | 41 | 0.26 |  | Y |  | PMID: 34311585 |
| Rv3132c | DevS | 5 | 162.1 | 37.9 | 0.23 | Y |  |  | PMID: 10970762 |
| Rv1368 | LprF | 4 | 166.8 | 33.2 | 0.2 |  |  | Y | PMID: 26201501 |
| Rv1555 | FrdD | 3 | 168.9 | 31.1 | 0.18 |  |  | Y | PMID: 18667562 |
| Rv1291c | MT1330 | 2 | 172.1 | 27.9 | 0.16 |  | Y |  |  |
| Rv0083 | MT0090 | 1 | 185.5 | 14.5 | 0.08 |  |  | Y |  |

*Note*: Rv-Exo abundance: the gene abundance in the H37Rv-treated exosome; Ra-Exo abundance: the gene abundance in the H37Ra-treated exosome; Ra-Exo/Rv-Exo ratio: the ratio between the gene abundance in the H37Ra-treated exosome and that in the H37Rv-treated exosome. VF, virulence factor.
