# Supplementary figures and images for: Macrophage Response to Avirulent and Virulent Mycobacterium tuberculosis and Anti-TB Effects of Exosome Treatment
Source: Genomics Proteomics Bioinformatics. 2025 Aug 5;23(6):qzaf065. doi: 10.1093/gpbjnl/qzaf065 (PMC13234453; doi:10.1093/gpbjnl/qzaf065)

**A**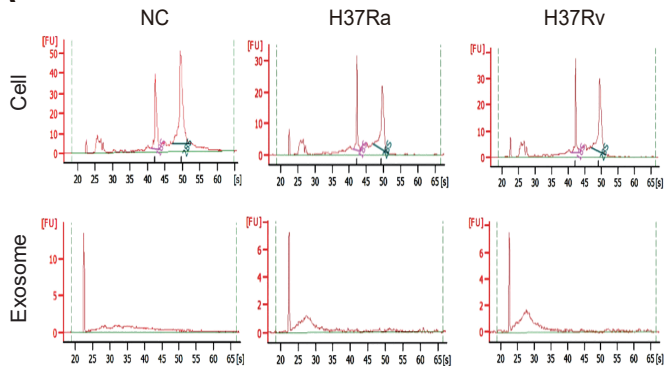**C**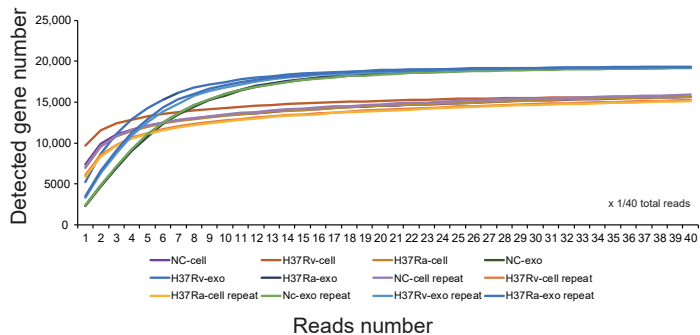**B**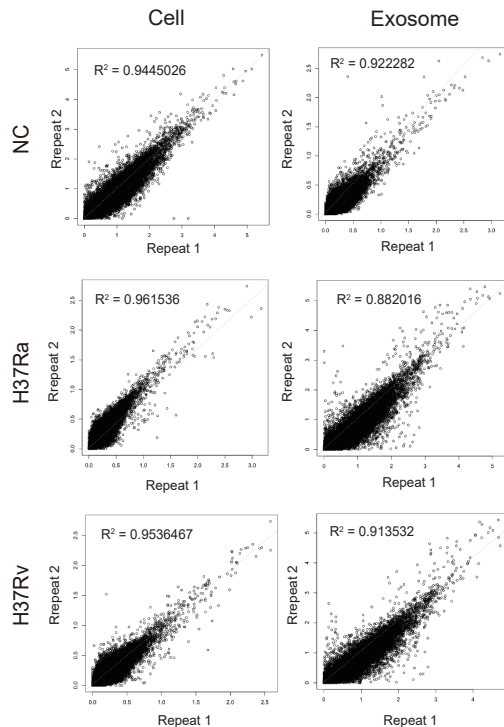

Supplement: qzaf065_Supplementary_Data [file qzaf065_supplementary_data.zip › Figure S1.pdf]

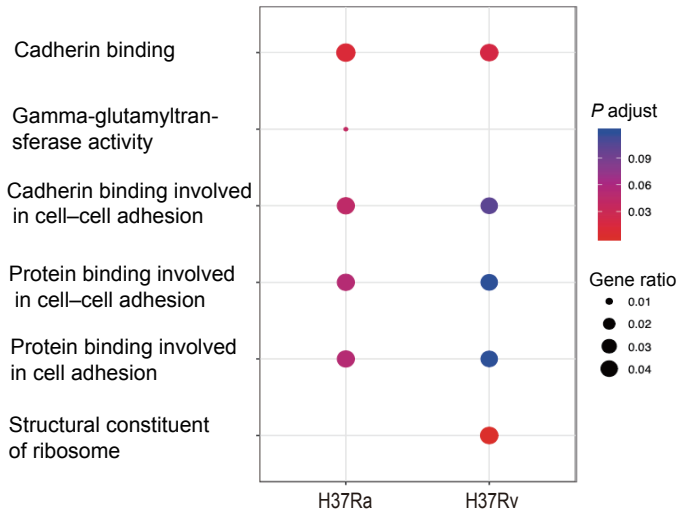

Supplement: qzaf065_Supplementary_Data [file qzaf065_supplementary_data.zip › Figure S2.pdf]

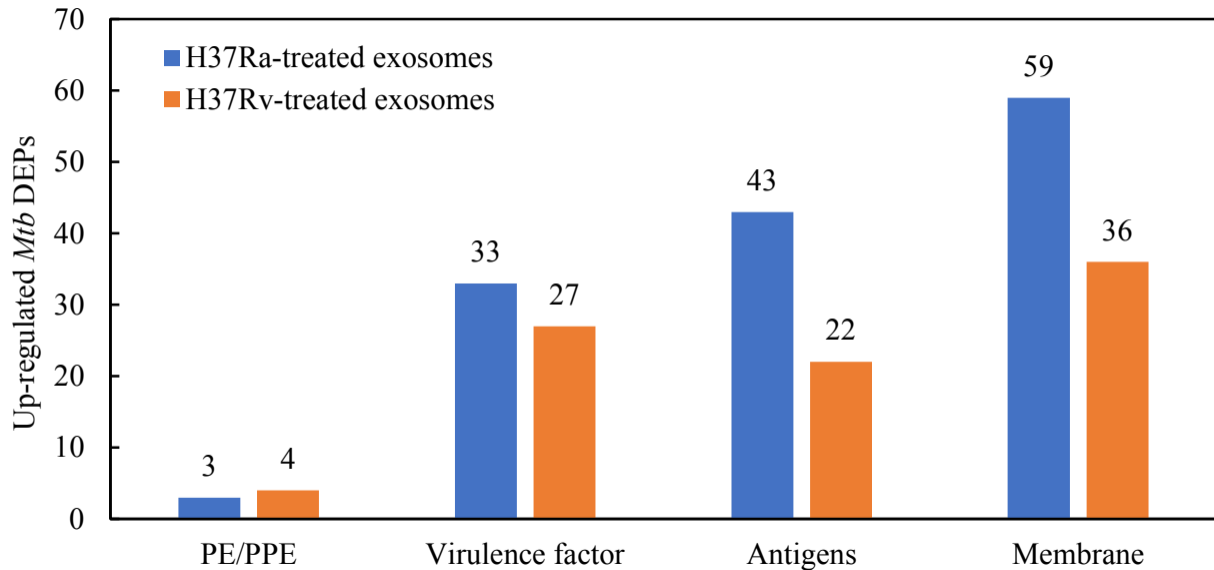

Supplement: qzaf065_Supplementary_Data [file qzaf065_supplementary_data.zip › Figure S3.pdf]

Transcriptome (DEGs)

Proteome (DEPs)

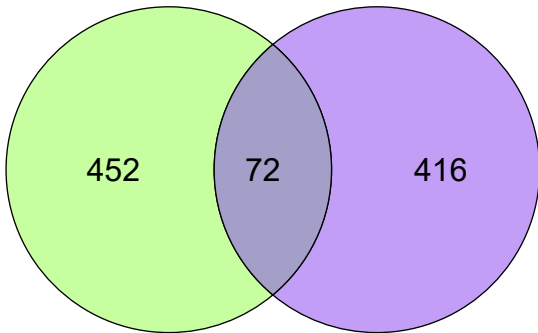

Supplement: qzaf065_Supplementary_Data [file qzaf065_supplementary_data.zip › Figure S4.pdf]

Anti-CD9

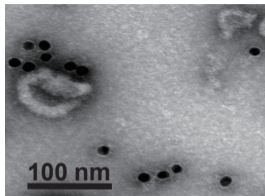

Anti-CD81

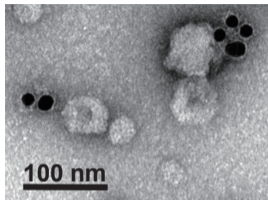

No-labeling

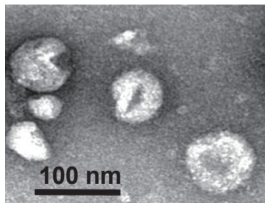

Anti-calreticulin

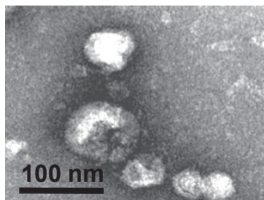

Supplement: qzaf065_Supplementary_Data [file qzaf065_supplementary_data.zip › Figure S5.pdf]
